# Supplementary material for: Immediate fluid management of children with severe febrile illness and signs of impaired circulation in low-income settings: a contextualised systematic review
Source: BMJ Open. 2014 Apr 30;4(4):e004934. doi: 10.1136/bmjopen-2014-004934 (PMC4010848; doi:10.1136/bmjopen-2014-004934)
Supplement: Author's manuscript [file bmjopen-2014-004934.draft_revisions.pdf]

Immediate fluid management of children with severe febrile illness and signs of impaired circulation in low income settings: a contextualized systematic review

Journal:	*BMJ Open*
Manuscript ID:	bmjopen-2014-004934
Article Type:	Research
Date Submitted by the Author:	25-Jan-2014
Complete List of Authors:	Opiyo, Newton ; Kenya Medical Research Institute/Wellcome Trust Research Programme, Health Services Unit<br>Molyneux, Elizabeth; College of Medicine and Queen Elizabeth Central Hospital, Department of Paediatrics<br>Sinclair, Dave; Liverpool School of Tropical Medicine, International Health Group<br>Garner, Paul; Liverpool School of Tropical Medicine, International Health Group<br>English, Mike; University of Oxford, Nuffield Department of Medicine and Department of Paediatrics
**Primary Subject Heading**:	Evidence based practice
Secondary Subject Heading:	Emergency medicine
Keywords:	Neonatal intensive & critical care < INTENSIVE & CRITICAL CARE, Paediatric intensive & critical care < INTENSIVE & CRITICAL CARE, PAEDIATRICS

Note: The following files were submitted by the author for peer review, but cannot be converted to PDF. You must view these files (e.g. movies) online.

Table_2_Study_inclusion_criteria.htm

SCHOLARONE™ Manuscripts

Immediate fluid management of children with severe febrile illness and signs of impaired circulation in low income settings: a contextualized systematic review

Newton Opiyo *Post-doctoral Researcher*[1*], Elizabeth Molyneux *Professor*[2], David Sinclair *Lecturer*[3], Paul Garner *Professor*[3], Mike English *Professor*[1, 4]

[1]Health Services Unit, KEMRI-Wellcome Trust Research Programme, Nairobi, 00100, Kenya; [2]Department of Paediatrics, College of Medicine and Queen Elizabeth Central Hospital, Blantyre, 3, Malawi; [3]International Health Group, Liverpool School of Tropical Medicine, Liverpool, L3 5QA, UK; [4]Nuffield Department of Medicine and Department of Paediatrics, University of Oxford, Oxford, OX3 7BN, UK

*** Corresponding author**

Newton Opiyo
KEMRI-Wellcome Trust Research Programme
P.O. Box 43640, Nairobi, 00100, Kenya
nopiyo@kemri-wellcome.org

**Word count**	Abstract	558
	Main text	2,858

For peer review only

For peer review only - http://bmjopen.bmj.com/site/about/guidelines.xhtml

Abstract

Objective

To evaluate the safety and effectiveness of fluid bolus (with either colloids or crystalloids) as part of immediate emergency care compared to maintenance fluids alone in children with severe febrile illness and signs of impaired circulation from a low income setting perspective.

Design

Systematic review of randomized controlled trials (RCTs), and observational studies, comparing fluid bolus with maintenance fluids alone. Our primary outcome measure was pre-discharge mortality.

Data sources and synthesis

We searched PubMed, The Cochrane Library, Google Scholar and Clinical Trial Registries up to March 2013. An updated MEDLINE search was done in January 2014. As studies used different clinical signs to define impaired circulation we classified patients into those with signs of severely impaired circulation, or those with any signs of impaired circulation. As the studies also used different fluid regimens, and incorporated different study designs, we did not pool data across studies. The quality of evidence for each outcome was appraised using the Grading of Recommendations Assessment, Development and Evaluation (GRADE) approach. Findings are presented as risk ratios (RRs) with 95% confidence intervals (CIs).

Results

Two RCTs and four observational studies were included. One RCT from district hospitals in East Africa recruited 3141 children; the remaining studies are smaller (34 to 187 participants) and all conducted in middle or high income settings with access to intensive care facilities.

Severely impaired circulation

The large RCT included a small subgroup that met the criteria for severely impaired circulation. Mortality was higher in those receiving bolus fluids (20-40 mls/kg/hr, saline or albumin) compared to maintenance fluids (2.5-4 mls/kg/hr) (RR 2.40, 95% CI 0.84 to 6.88, P=0.054, 65 participants, *low quality evidence*). Three additional observational studies in this group were at high risk of confounding and found mixed effects (*very low quality evidence*).

Any signs of impaired circulation

The RCT from East Africa included children with signs of both severely and non-severely impaired circulation. Overall, bolus fluids increased 48-hour mortality compared to maintenance fluids with an additional 3 deaths per 100 children treated (RR 1.45, 95% CI 1.13 to 1.86, 3141 participants, *high quality evidence*). In a second small RCT from India, no difference in 72-hour mortality was detected between children who received 20-40 mls/kg Ringers lactate over 15 minutes and those who received

For peer review only - http://bmjopen.bmj.com/site/about/guidelines.xhtml

20 mls over 20 minutes up to a maximum of 60 mls/kg over 1 hour (147 participants, *low quality evidence*).

In one additional observational study, resuscitation consistent with APLS guidelines, including fluids, was not associated with reduced mortality in the small sub-group with septic shock (*very low quality evidence*).

Signs of impaired circulation, but not severely impaired

Only the large RCT from East Africa allowed an analysis for children with some signs of impaired circulation who would not meet the criteria for severe impairment. Bolus fluids increased 48-hour mortality compared to maintenance alone (RR 1.36, 95% CI 1.05 to 1.76, *high quality evidence*).

Conclusion

Prior to the publication of the large multi-centre RCT from East Africa, the evidence for bolus fluid therapy in children with severe febrile illness and signs of impaired circulation was of very low quality, and poorly applicable to low resource settings. This large study provides robust evidence that in low resource settings fluid boluses increase mortality in children with severe febrile illness and impaired circulation, and this increased risk is consistent across children with severe and less severe circulatory impairment.

Keywords: Febrile illness, fluid resuscitation, shock, children, systematic review

For peer review only

For peer review only - http://bmjopen.bmj.com/site/about/guidelines.xhtml

Strengths and limitations of this study

- Timely systematic review given the current uncertainty on fluid resuscitation in children; review incorporates data on the largest randomised controlled trial (FEAST trial) on fluid therapy in paediatric emergency care;
- Review includes a comprehensive search strategy, and uniquely uses the robust GRADE methodology to synthesize available evidence;
- Review limitations include small number of studies and observational studies with small sample sizes.

For peer review only

For peer review only - http://bmjopen.bmj.com/site/about/guidelines.xhtml

Background

Severely ill children require health staff to rapidly assess, resuscitate and treat them to prevent death. Practical, simple to use protocols have been established to guide care in an often stressful, busy environment. In North America and Europe, the most commonly used are the Pediatric Advanced Life Support (PALS)[1] and European Paediatric Life Support (EPLS)[2] guidelines respectively.

The World Health Organization have specific guidelines for low income settings without intensive care facilities called the Emergency Triage Assessment and Treatment (ETAT) guidelines. ETAT guidance begins with initial triage to identify children who need urgent formal assessment such as altered consciousness or severe breathing or circulation problems. Immediate care should then follow an ABCD approach to assessment and action which prioritises care of the airway (A) first, followed by breathing (B) and circulation (C).

Septic shock

Severe febrile illness in children is often associated with signs of impaired circulation (also known as 'septic shock'). Current ETAT guidelines define shock as the presence of **three** clinical signs of poor peripheral perfusion: weak / absent peripheral pulse, prolonged capillary refilling >3 seconds, **and** cold hands and feet (typically with cold skin extending up the limb and termed 'a temperature gradient'). International guidance, including ETAT, typically recommends a rapid fluid bolus of 20 to 40 mls/kg intravenously once 'shock' is diagnosed.[3]

Why it is important to do this review

As early as 1999, authors were arguing that no robust data existed demonstrating that bolus fluid resuscitation improved clinical outcomes in septic patients.[4] Moreover, the results of observational studies suggested that large volumes of resuscitation fluids may be associated with increased morbidity in patients with sepsis[5], acute respiratory distress syndrome[6] or acute kidney injury.[7]

Subsequently, a large, multicentre, randomized controlled trial published in 2011, and conducted in East Africa found that bolus fluid resuscitation increased mortality compared to a maintenance fluid regimen in children with 'febrile illness' and impaired perfusion.[8] The results of this trial clashed with the long-standing belief that fluid bolus resuscitation was beneficial and caused considerable international debate.[9-12] To date, no clear guidance has emerged on how to incorporate these findings into recommendations for practice in high or low income settings.

Objective

To evaluate the safety and effectiveness of fluid bolus (with either colloids or crystalloids) compared to maintenance fluids alone in children with severe febrile illness and signs of impaired circulation.

Methods

Criteria for considering studies for this review

For peer review only - http://bmjopen.bmj.com/site/about/guidelines.xhtml

Types of studies: Randomised controlled trials (RCTs), quasi-experimental studies, controlled before and after studies and cohort studies.

Types of participants: Children (aged ≤18 years) with clinical features suggesting impaired perfusion (including shock) due to presumed acute infectious illnesses or inflammatory state (sepsis) and excluding diarrhoeal illness (as defined by the studies).

Types of interventions: Intravenous fluid boluses (crystalloids or colloids) compared to no (or lower volume) fluid boluses or maintenance fluids.

Types of outcome measures: The primary outcome measure was pre-discharge mortality. Secondary outcomes were: mortality at any time up to 4 weeks and adverse clinical events reported in the studies.

Data sources and search strategy

We searched PubMed, The Cochrane Library, Google Scholar and Clinical Trial Registries (ClinicalTrials.gov, Current Controlled Trials, WHO International Clinical Trials Registry Platform, metaRegister of Controlled Trials) for eligible published, unpublished or in-progress articles. The searches covered the period from the databases start dates to March 2013. An updated MEDLINE search was done in January 2014. No date or language restrictions were used.

The searches were performed iteratively by combining MeSH (Medical Subject Headings) and free text terms relevant to the conditions (sepsis, septicemia, febrile illness, bacteremia, infection, meningitis, septic shock, hypovolemia), treatments (fluids, resuscitation, intravenous fluids, fluid therapy) and patient groups (neonates, infants, children, adolescents) of interest. No date or language restrictions were used.

In addition we searched the websites of relevant organizations (the International Sepsis Forum, the World Federation of Pediatric Intensive Care and Critical Care Societies) and key emergency / intensive care journals (Critical Care Medicine, Critical Care, Pediatric Emergency Medicine, Shock, Resuscitation, Intensive Care Medicine). Reference lists of related systematic reviews and primary studies were handsearched. We also sought additional papers by contacting authors of related reviews and selected studies.

Study selection

Two reviewers (NO and ME) independently screened the titles, abstracts and full texts of retrieved articles and applied the study eligibility criteria detailed above to select studies. Disagreements were resolved by discussion.

Data extraction

For peer review only

For peer review only - http://bmjopen.bmj.com/site/about/guidelines.xhtml

Data were extracted using a pre-designed form by one reviewer (NO) and checked by the other reviewers; disagreements were resolved by discussion. We extracted data on: study designs, settings, sample size, participants (diagnoses, age range), shock definitions, treatments and comparisons (types of fluids, timing, volumes and fluid rates), co-interventions and proportion of patients experiencing the events of interest in each treatment group.

Risk of bias in individual studies

Two reviewers (NO, DS) independently assessed the risk of bias in the included studies according to six criteria assessing the risk of selection bias (random sequence generation, allocation concealment, selection of two groups), reporting bias (blinding), and confounding (baseline characteristics, co-interventions). For each criteria, the study was classified as high risk of bias, low risk of bias or unclear risk of bias.

Assessment of quality of evidence

The quality of evidence for each of the efficacy and safety outcomes was assessed using the GRADE (Grading of Recommendation Assessment Development and Evaluation) approach.[13] Key quality elements assessed by GRADE include: risk of bias (study limitations), precision of treatment effects, consistency of results, directness (applicability) of evidence, and publication bias. The GRADE evidence profiles were prepared by one reviewer (NO) and verified independently by two reviewers (ME, DS). Discrepancies in the quality ratings were resolved by discussion.

Synthesis of results

We summarized results narratively due to significant differences in study designs, fluid protocols and patient risk profiles. In order to compare studies with similar populations we grouped studies by the severity of circulatory impairment at baseline, after an appraisal of the clinical signs used to define inclusion. We defined three groups:

1. Severely impaired circulation (SIC): Studies where inclusion criteria were similar to the ETAT guidance (shock defined as presence of all three signs of impaired circulation).
2. Impaired circulation (IC): Studies where inclusion only required one or two of these signs.
3. Impaired circulation but without severe impairment: Studies where inclusion required one or two signs of impaired circulation and more severely ill patients were excluded.

For consistency all results are presented as risk ratios (RRs) with 95% confidence intervals (CIs) (where reported, odds ratios and percentage point differences were converted into risk ratios).

Results

Study selection process

For peer review only - http://bmjopen.bmj.com/site/about/guidelines.xhtml

For peer review only

The flow of studies through this review is summarized in Figure 1. Six studies fulfilled all our pre-specified eligibility criteria: two randomized controlled trials[8,14], two prospective cohort studies[15,16], and two retrospective record reviews.[17,18]

Study characteristics

The characteristics of the six included studies are summarized in table 1. Study settings were varied: USA[15-17], Brazil[18], India[14] and East Africa (Kenya, Tanzania and Uganda).[8] Five studies[14-18] were conducted in settings where paediatric intensive care unit facilities including inotropic support, intubation and ventilation were available. The largest study[8], was conducted in typical resource-limited East African hospitals without these additional measures being available. The study sample sizes ranged from 34 to 3,141 patients.

The clinical definitions of severe febrile illness and circulatory impairment or ‘shock’ varied across studies (table 2). Fluid therapy protocols (volumes, timing, rates) were similarly varied between comparison study groups (table 1). In only one case, the work conducted in East Africa, was a clearly defined ‘no bolus’ group studied.[8]

Risk of bias

Both RCTs were considered to be of low risk of bias (table 3). The observational studies were all potentially confounded (none met all the risk of bias criteria; table 3). The following key criteria were only partially met or were unclear from the information provided: appropriate participant selection, study power, appropriate blinding/outcome assessments, and association of outcome and treatment.

Efficacy and safety outcomes

The results for the efficacy and safety outcomes, together with quality of evidence, are summarized in the following three distinct population risk groups.

(a) Children with severe febrile illness and signs of severely impaired circulation (SIC)

Four studies reported outcome data in children relevant to those with severely impaired circulation: one RCT[8], one prospective cohort[16], and two retrospective studies.[16,18] Overall mortality in the patients reported in these analyses were: 42%, 51%, 29% and 47% respectively.

Trial data: The RCT[8] included children with milder forms of circulatory impairment but provided a sub-group analysis of 65 patients who fulfilled the ETAT criteria of ‘shock’. In this subgroup a fluid bolus of 20 to 40 mls/kg (saline or albumin) over 1 hour was associated with a considerably higher risk of mortality than maintenance fluids (2 to 4 mls/kg/hr) but this was of borderline statistical significance (RR 2.40, 95% CI, 0.84 to 6.88, P=0.054, *low quality evidence*; table 4).

Observational data: In Brazil a retrospective records review of 90 children admitted to a paediatric intensive care unit with sepsis and shock assessed the relationship between mortality and fluid resuscitation in the first hour.[18] Administration of 20 mls/kg of bolus resuscitation fluid (crystalloids,

For peer review only - http://bmjopen.bmj.com/site/about/guidelines.xhtml

colloids) was associated with a significantly higher mortality than if 40 mls/kg or more was given (73% versus 33%, RR 0.45, 95% CI not estimable, P<0.05, *very low quality evidence*; table 4). Of particular note 80 of the 90 children studied had severe, pre-existing chronic disease such as malignancy.

A second retrospective records review from the USA, examined mortality in 91 children being resuscitated and referred by community physicians.[17] In this study 'appropriate fluid therapy' was reported to be strongly associated with improved survival (RR 0.20, 95% CI 0.05 to 0.78, *very low quality evidence*; table 4). However, the 'appropriate fluid therapy' group included those with fluids given as per guidelines and children in whom signs of shock resolved quickly irrespective of the volumes of fluid given. In fact, the median fluid volumes given to children who died (32.9 mls/kg) were higher than those given to survivors (20 mls/kg).

The third study, a prospective cohort from the USA, included 34 children admitted to the paediatric intensive care unit with microbiologically proven septic shock, and all were receiving inotropes and had a pulmonary catheter inserted.[16] Those receiving less than 20 mls/kg of fluids during the first hour of resuscitation (normal saline, Ringers lactate, 5% albumin) had significantly higher mortality than those given 20 to 40 mls/kg of fluids (RR 1.11, 95% CI 0.59 to 2.11, *very low quality evidence*) or more than 40 mls/kg (RR 0.19, 95% CI 0.03 to 1.30, *very low quality evidence*; table 4).

(b) Children with severe febrile illness and any sign of impaired circulation (IC)

Two randomized controlled trials[8,14] and one prospective cohort study[15] enrolled febrile children with clinical signs sufficiently similar to be included in the impaired circulation (IC) category. Overall mortality in the patients reported in these studies were 9.5%, 18% and 12.5% respectively.

Trial data: In the largest RCT from Africa 3,141 children were randomized to a fluid bolus (20 to 40 mls/kg albumin or normal saline over 1 hour) or maintenance fluids (2.5 to 4.0 mls/kg/hour).[8] Compared to maintenance fluids, the fluid bolus was associated with increased 48-hour mortality (RR 1.45, 95% CI 1.13 to 1.86, *high quality evidence*; table 5) and increased mortality at 4 weeks (RR 1.39, 95% CI 1.11 to 1.74, *moderate quality evidence*; table 5). There was no suggestion that albumin performed any differently than saline (albumin bolus vs. saline bolus, RR 1.0, 95% CI 0.78 to 1.29). There was no difference between the bolus and maintenance fluid groups in the risk of neurologic sequelae at 4 weeks (RR 1.03, 95% CI 0.61 to 1.75, *low quality evidence*; table 5) or the combined outcome of pulmonary edema or increased intracranial pressure (RR 1.46, 95% CI 0.85 to 2.53, *low quality evidence*; table 5). A subgroup analysis suggested that the increased mortality was only statistically significant in children with severe anaemia (hemoglobin <5 g/dl) (RR 1.71, 95% CI 1.16 to 2.51, *moderate quality evidence*; table 5), but a subsequent analysis exploring the effect of anaemia when treated as a continuous variable found evidence of harm with fluid bolus across the full range of haemoglobin values.[19]

In the second RCT, 147 children in an Indian paediatric intensive care unit were randomized to receive Ringers lactate 20 to 40 mls/kg over 15 minutes or Ringers lactate 20 mls/kg over 20 minutes up to a maximum of 60 mls/kg over 1 hour.[14] There was no difference in 72-hour mortality between comparison groups (RR 0.99, 95% CI 0.49 to 1.98, *low quality evidence*; table 5).

For peer review only - http://bmjopen.bmj.com/site/about/guidelines.xhtml

Observational data: The prospective cohort study included 1,409 children but only 187 had septic shock and so were relevant to this review.[15] No difference in mortality was observed in this subgroup between those receiving resuscitation consistent with Pediatric Advanced Life Support (PALS)/Advanced Pediatric Life Support (APLS) recommendations (including rapid bolus 20 mls/kg of isotonic fluid, potentially repeated and use of inotropes) performed by community physicians compared to resuscitation not consistent with these recommendations (*very low quality evidence*; table 5). In addition, the actual fluid volumes administered and the relationship between fluid volumes given and outcomes is not presented. Furthermore, resuscitation episodes were classified as having been consistent with guidelines if signs of shock resolved early in the course of intervention, irrespective of actual fluid volumes given.

(c) Children with severe febrile illness and impaired circulation but not severely impaired circulation (IC but not SIC)

It was possible to derive outcome data for children with severe febrile illness and any sign of circulatory impairment (IC) but not severely impaired circulation (SIC) in one study[20]: 20 to 40 mls/kg bolus fluids (albumin, normal saline) provided over 1 hour, compared to 2.5 to 4.0 mls/kg/hr maintenance fluids, was associated in this large sub-group (n=3,076 children) with increased 48-hour mortality (RR 1.36, 95% CI 1.05 to 1.76, *high quality evidence*).

Discussion

This review was conducted to facilitate revision of national paediatric fluid management guidelines in Kenya and potentially neighboring countries using ETAT guidance, but has direct policy implications for rural health care across Africa, and even for those involved in paediatric care in high resource settings.

The limited data available prior to the FEAST study demonstrate that the current recommendations for fluid bolus included in the ETAT guidelines were not supported by a strong scientific evidence base. In fact, the evidence was largely observational, and from intensive care settings not directly comparable to typical resource-poor settings in Africa. Added to this, the studies were themselves flawed, with design aspects which potentially biased the result towards favouring high volume fluid resuscitation. Of particular note are two of the retrospective studies which classified all children who recovered quickly as having received 'adequate fluid therapy' irrespective of the fluid volume they received. The potential for bias in these studies resulting from possible exposure misclassification should be noted by paediatricians working in high resource settings.

The RCT published in 2011 provides by far the most direct assessment of fluid boluses in children with severe febrile illness in resource poor African settings that do not typically see Dengue Fever but where malaria may be common.[8] In these settings emergency management decisions must typically be made without accurate blood pressure reading or investigations such as pulse oximetry, blood gas analysis, haemoglobin or lactate measurement. The robust finding of increased mortality in the large group of children with an initial, clinical diagnosis of severe febrile illness and impaired circulation but not severely impaired circulation demonstrates that the clinical signs linked to this classification are, alone, not sufficient to identify children in whom fluid boluses may be beneficial. In fact this trial provides robust evidence that bolus intravenous fluids are harmful in such children.

For peer review only - http://bmjopen.bmj.com/site/about/guidelines.xhtml

Data from the small sub-group of children within the FEAST study who had all three clinical signs of severely impaired circulation, are the only randomized evidence on the risks and benefits of fluid boluses for this group. Mortality amongst those receiving bolus fluids was higher than those receiving maintenance fluids but the data are compatible with an effect ranging from a small potential benefit of bolus to very substantial harm, which raises severe doubt about the use of boluses even in this more severely ill group.

In an effort to accommodate the highly influential observational research on fluid use in paediatric emergency care we included data from studies traditionally excluded from systematic reviews (such as the recent review by Ford and colleagues[21]). Although this presented challenges it is an advantage of the GRADE approach that the quality of evidence from such studies can be transparently appraised and, potentially therefore, contribute to informed decision making.

Conclusion

Prior to the publication of the large multicentre African RCT, the evidence in support of aggressive fluid therapy in children with septic shock was only of very low quality, and poorly applicable to low resource settings. The 2011 RCT provides robust evidence that in low resource settings fluid boluses, even in relatively small amounts, increase mortality in children with severe febrile illness and signs of impaired circulation. For children with signs of severely impaired circulation the evidence suggests harm but there is less certainty and further research is warranted.

For peer review only - http://bmjopen.bmj.com/site/about/guidelines.xhtml

For peer review only

Copyright

The Corresponding Author has the right to grant on behalf of all authors and does grant on behalf of all authors, an exclusive licence (or non exclusive for government employees) on a worldwide basis to the BMJ Publishing Group Ltd to permit this article (if accepted) to be published in BMJ editions and any other BMJPGL products and sublicences such use and exploit all subsidiary rights, as set out in our licence.

Competing interests

All authors have completed the Unified Competing Interest form atwww.icmje.org/coi_disclosure.pdf (available on request from the corresponding author) and declare: no support from any organisation for the submitted work; no financial relationships with any organisations that might have an interest in the submitted work in the previous three years, no other relationships or activities that could appear to have influenced the submitted work.

Elizabeth Molyneux worked with WHO to develop the initial ETAT training and supports the provision of ETAT training in Malawi and other countries. Mike English adapted ETAT when creating ETAT+ and supports the provision of ETAT+ training in Kenya and has facilitated use of ETAT+ in other countries.

Authors' contributions

NO conducted the searches. NO, DS and ME screened records for eligibility, extracted data, assessed study quality and analysed data. EM and PG reviewed selected studies. NO wrote the first draft of the manuscript. All the authors participated in the interpretation of results and writing of the full manuscript. NO and ME are the guarantors.

Ethical approval: Not required.

Funding

Newton Opiyo is funded by a post-doctoral training grant from the Consortium for National Health Research. Mike English is supported by a Wellcome Trust Senior Fellowship (#097170). Additional funds from a Wellcome Trust Strategic Award (#084538) and a Wellcome Trust core grant awarded to the KEMRI-Wellcome Trust Research Programme (#092654) made this work possible. The guideline development meeting was support in part by the Effective Health Care Research Consortium, which is funded by UKaid from the UK Government Department for International Development. The funding source had no role in study design, data collection, analysis, data interpretation, writing of the report or the decision to submit for publication. All authors carried out this research independently of the funding body.

Data sharing: No additional data available.

For peer review only - http://bmjopen.bmj.com/site/about/guidelines.xhtml

References

1. American Heart Association. 2005 American Heart Association (AHA) guidelines for cardiopulmonary resuscitation (CPR) and emergency cardiovascular care (ECC) of pediatric and neonatal patients: pediatric basic life support. Pediatrics 2006;117(5):e989-1004
2. Phillips B, Mackway-Jones K, Jewkes F. The European Resuscitation Council's paediatric life support course 'Advanced Paediatric Life Support'. Resuscitation 2000;47(3):329-334
3. Brierley J, Carcillo J, Choong K, *et al*. Clinical practice parameters for hemodynamic support of pediatric and neonatal septic shock: 2007 update from the American College of Critical Care Medicine. Crit Care Med 2009;37(2):666-88. Erratum in: Crit Care Med. 2009 Apr;37(4):1536.
4. Jardin F, Fourme T, Page B, *et al*. Persistent preload defect in severe sepsis despite fluid loading: A longitudinal echocardiographic study in patients with septic shock. Chest 1999;116(5):1354-9
5. Durairaj L, Schmidt G. Fluid therapy in resuscitated sepsis: less is more. Chest 2008;133(1):252-63
6. Wiedemann H. A perspective on the fluids and catheters treatment trial (FACTT). Fluid restriction is superior in acute lung injury and ARDS. Cleve Clin J Med 2008;75(1):42-8
7. Prowle J, Echeverri J, Ligabo E, *et al*. Fluid balance and acute kidney injury. Nat Rev Nephrol 2010;6(2):107-15
8. Maitland K, Kiguli S, Opoka R, *et al*. Mortality after fluid bolus in African children with severe infection. N Engl J Med 2011;364(26):2483-95
9. Brewster D. African fluid bolus study: implications for practice J Paediatr Child Health 2012;48(7):548-50
10. Duke T. What the African fluid-bolus trial means. Lancet 2011;378(9804):1685-7
11. Hilton A, Bellomo R. A critique of fluid bolus resuscitation in severe sepsis. Crit Care 2012;16(1):302
12. Southall D, Samuels M. Treating the wrong children with fluids will cause harm: response to 'mortality after fluid bolus in African children with severe infection'. Arch Dis Child 2011;96(10):905-6
13. GRADE Working Group. Available at: http://www.gradeworkinggroup.org/.
14. Santhanam I, Sangareddi S, Venkataraman S, *et al.* A prospective randomized controlled study of two fluid regimens in the initial management of septic shock in the emergency department. Pediatr Emerg Care 2008;24(10):647-55
15. Carcillo J, Kuch B, Han Y, *et al*. Mortality and functional morbidity after use of PALS/APLS by community physicians. Pediatrics 2009;124(2):500-8
16. Carcillo J, Davis A, Zaritsky A. Role of early fluid resuscitation in pediatric septic shock. JAMA 1991;266(9):1242-5
17. Han Y, Carcillo J, Dragotta M, *et al*. Early reversal of pediatric-neonatal septic shock by community physicians is associated with improved outcome. Pediatrics 2003;112(4):793-9
18. Oliveira C, Nogueira dSF, Oliveira D, *et al*. Time- and fluid-sensitive resuscitation for hemodynamic support of children in septic shock: barriers to the implementation of the American College of Critical Care Medicine/Pediatric Advanced Life Support Guidelines in a pediatric intensive care unit in a developing world. Pediatr Emerg Care 2008;24(12):810-5
19. Maitland K, George E, Evans J, *et al*. Exploring mechanisms of excess mortality with early fluid resuscitation: insights from the FEAST trial. BMC Med 2013;11:68
20. Maitland K, Akech S, Russel E. Mortality after fluid bolus in African children with sepsis (Author's response). N Engl J Med 2011b;365(14):1351-1353
21. Ford N, Hargreaves S, Shanks L. Mortality after fluid bolus in children with shock due to sepsis or severe infection: a systematic review and meta-analysis. PLoS One 2012;7(8):e43953

For peer review only - http://bmjopen.bmj.com/site/about/guidelines.xhtml

Figure Legends

Figure 1. Results of literature search and studies selected

Table Legends

Table 1. Characteristics of included studies

Table 2. Study inclusion criteria

Table 3. Study quality assessment

Table 4 and 5. Summary of findings and quality of evidence

For peer review only

For peer review only - http://bmjopen.bmj.com/site/about/guidelines.xhtml

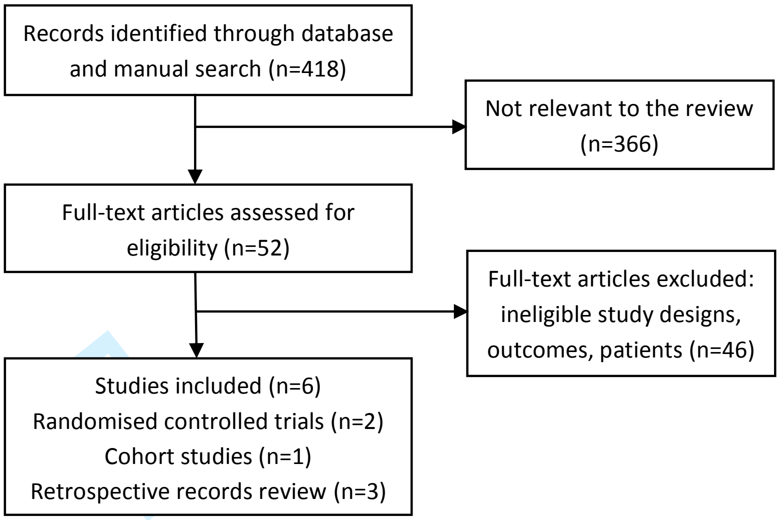

Figure 1. Results of literature search and studies selected

For peer review only

Table 1. Characteristics of included studies

Study	Study design	Setting	Number of children	Intervention	Control	Co-interventions
Maitland et al [12]	RCT	Kenya Tanzania Uganda	3,141	20 to 40 mls/kg of 5% albumin or 0.9% saline over the first hour, followed by maintenance fluids	Intravenous maintenance fluids alone (2.5 to 4.0 mls/kg/hr)	Antibiotics, antimalarials, antipyretics, anticonvulant drugs, hypoglycemia treatment and blood transfusion
Santhanam et al [18]	RCT	India	147	20 to 40 mls/kg of Ringers lactate over 15 minutes followed by dopamine if therapeutic goal not achieved after 15 minutes	20 mls/kg over 20 minutes up to 60 mls/kg over 1 hour followed by dopamine if therapeutic goal not achieved at 1 hour	Antibiotics, anticonvulsant drugs, intubation, ventilation support, treatment of hypoglycemia, hypocalcemia and asthma
Carcillo et al [19]	Prospective cohort	USA	187 (a subgroup)	Received PALS/APLS - recommended resuscitation (20 to 40 mls/kg) or recovered quickly	Did not receive PALS/APLS - recommended resuscitation	Inotropic therapy
Han et al [20]	Retrospective records review	USA	91	Received PALS/APLS recommended resuscitation (>60 mls/kg normal saline) or recovered	Did not receive PALS/APLS recommended resuscitation	Antibiotics, inotropic/vasopressor support and hydrocortisone therapy
Carcillo et al [21]	Prospective cohort	USA	34	> 40 mls/kg total fluids (normal saline, Ringers lactate, 5% albumin or blood products)	<20 mls/kg total fluids or 20 to 40 mls/kg total fluids	Antibiotics, vasopressor and/or inotropic support and assisted ventilation
Oliveira et al [22]	Retrospective records review	Brazil	90	>40 mls/kg in the first hour (colloids, crystalloids, or packed red blood cells)	<20 mls/kg in the first hour	Antibiotics and inotropic support

For peer review only

Table 1. Characteristics of included studies (continued)

Study	Study design	Definition of severe febrile illness	Definition of circulatory impairment	Our classification	Overall mortality
Maitland 2011 [12]	Randomized controlled trial	Febrile illness with impaired consciousness and/or respiratory distress	One or more of: capillary refilling time ≥3 secs, severe tachycardia, temperature gradient or weak pulse	Some circulatory impairment (IC)<br><br>(Subgroup of severely impaired circulation (SIC))	9.5%<br><br>(42%)
Santhanam 2008 [18]	Randomized controlled trial	Children with septic shock	Tachycardia with signs of decreased perfusion including: decreased peripheral pulses compared with central pulses, altered alertness, flash capillary refill or capillary refill >2 secs, mottled or cool extremities, or decreased urine output	Some circulatory impairment (IC)	18%
Carcillo et al [19]	Prospective cohort	Infants and children with trauma and non-trauma diagnoses (including septic shock)	Prolonged capillary refill time (>3 seconds or mottled extremities) and/or hypotension (systolic blood pressure less than the fifth percentile for age according to PALS/APLS criteria)	Some circulatory impairment (IC)	12.5%
Han et al [20]	Retrospective records review	Infants and children with septic shock	Decreased perfusion, including decreased mental status, prolonged capillary refill time (>3 seconds), diminished peripheral pulses, or mottled extremities and hypotension (systolic blood pressure less than the fifth percentile for age)	Severe circulatory impairment (SIC)	29%
Carcillo et al [21]	Prospective cohort	Children with septic shock	Blood pressure <2 SDs below the mean for age, combined with three of the following four criteria for decreased perfusion: decreased peripheral pulses; mottled or cool extremities; tachycardia (heart rate >180 bpm for patients <5 years of age; and >160 bpm for patients ≥5 years of age); or urine output <1 mls/kg per hour (or <20 mls/hr in children weighing >20 kg)	Severe circulatory impairment (SIC)	51%
Oliveira et al [22]	Retrospective	Children with sepsis and	Decreased perfusion (decreased peripheral pulses,	Severe circulatory	47%

For peer review only

	records review	septic shock	mottled or cool extremities, capillary refill time <1 second or >3 seconds), hypotension (systolic blood pressure less than the fifth percentile for age, using the PALS formula), altered mental status, or oliguria (<1 mls/kg per hour)	impairment (SIC)

bpm: Beats per minute; PALS: Pediatric Advanced Life Support; APLS: Advanced Pediatric Life Support; SD: Standard deviation;
IC: Severe febrile illness and any sign of impaired circulation;
SIC: Severe febrile illness and signs of severely impaired circulation;
IC but not SIC: Severe febrile illness and impaired circulation but not severely impaired circulation

For peer review only

Table 3. Quality assessment of included studies

**Study ID**	**Study design**	**Selection bias**			**Reporting Bias**	**Confounding**	
		Random sequence generation	Allocation concealment	Selection of two groups	Blinding	Baseline characteristics	Co-interventions
**Maitland 2011**	Randomized controlled trial	Low risk	Low risk	Low risk	Low risk	Low risk	Low risk
**Santhanam 2008**	Randomized controlled trial	Low risk	Low risk	Low risk	Low risk	Low risk	Low risk
**Carcillo 2009**	Prospective cohort	N/A	N/A	High risk[1]	N/A	High risk[1]	Unclear risk
**Oliveira 2008**	Retrospective records review	N/A	N/A	High risk[2]	N/A	High risk[2]	Unclear risk
**Han 2003**	Retrospective records review	N/A	N/A	High risk[3]	N/A	High Risk[3]	Unclear risk
**Carcillo 1991**	Prospective cohort	N/A	N/A	Low risk	N/A	Unclear risk	Unclear risk

[1] Carcillo 2009: Compared those who received recommended APLS/PALS treatment with those who didn't. 'Received recommended APLS/PALS fluid therapy' was defined as those who recovered regardless of fluid therapy plus those who didn't recover but received >20 mg/kg of fluids. Children who didn't receive recommended APLS/PALS treatment were significantly younger and had significantly longer capillary refill times, lower blood pressure, and higher oxygen requirements.

[2] Oliviera 2008: Compared survivors and non-survivors. The survivors were more likely to have had higher fluid volumes but were also significantly younger.

[3] Han 2003: Compared survivors and non-survivors. Appropriate fluid therapy was defined as those who recovered regardless of fluid therapy plus those who didn't recover but received >60 mg/kg of fluids. Non-survivors had significantly higher PRISM scores at baseline (PRISM assesses the risk of mortality).

[4] Carcillo 1991: Compared those who received three different fluid regimens. Adequate baseline characteristics were not presented.

For peer review only

Table 4. Summary of findings and quality of evidence

Bolus fluids compared to maintenance fluids alone in children with severe febrile illness and severely impaired circulation (SIC)

Patient or population: Children with severe febrile illness and severely impaired circulation
Settings: Hospitals (East Africa, USA, Brazil)
Intervention: Bolus fluid resuscitation (40 mls/kg albumin or normal saline) followed by maintenance fluids (2.5 to 4.0 mls/kg)
Comparison: Maintenance fluids alone (2.5 to 4.0 mls/kg)

Outcomes	Study design	Illustrative comparative risks* (95% CI)		Relative effect (95% CI)	No of Participants (studies)	Quality of the evidence (GRADE)	Comments
		Assumed risk	Corresponding risk				
		Maintenance fluids alone	Bolus followed by maintenance fluids				
**Mortality**	RCT	**20 per 100**	**48 per 100** (17 to 138)	**RR 2.40** (0.84 to 6.88)	65 (1 study)	⊕⊕⊖⊖ **low**[1,2,3,4]	Maitland et al [12]
	Prospective cohort	**60 per 100**	**11 per 100** (2 to 73)	**RR 0.19** (0.03 to 1.21)	34 (1 study)	⊕⊖⊖⊖ **very low**[10,11]	Carcillo et al [21]
	Retrospective cohort	**73 per 100**	**33 per 100** (NA)	**RR 0.45** (NA)	90 (1 study)	⊕⊖⊖⊖ **very low**[5,6,7]	Oliveira et al [22]
	Retrospective cohort	**38 per 100**	**8 per 100** (2 to 30)	**RR 0.20** (0.05 to 0.78)	91 (1 study)	⊕⊖⊖⊖ **very low**[8,9]	Han et al [20]

*The basis for the **assumed risk** (e.g. the median control group risk across studies) is provided in footnotes. The **corresponding risk** (and its 95% confidence interval) is based on the assumed risk in the comparison group and the **relative effect** of the intervention (and its 95% CI).
CI: Confidence interval; **RR:** Risk ratio

GRADE Working Group grades of evidence
High quality: Further research is very unlikely to change our confidence in the estimate of effect.
Moderate quality: Further research is likely to have an important impact on our confidence in the estimate of effect and may change the estimate.
Low quality: Further research is very likely to have an important impact on our confidence in the estimate of effect and is likely to change the estimate.
Very low quality: We are very uncertain about the estimate.

[1] This data represents a sub-group of patients from the large FEAST trial in East Africa [12]. These 65 patients had signs of a febrile illness and fulfilled the ETAT definition of shock.
[2] No serious risk of bias: Randomization and allocation concealment were adequate to reduce the risk of selection bias. Study staff were un-blinded to the intervention.
[3] Downgraded by 1 for serious indirectness: Children with severe hypotension were excluded. The trial was conducted in hospital settings in Kenya, Tanzania and Uganda. Children were aged 2 months to 12 years. Children with severe malnutrition, gastroenteritis, or shock due to trauma surgery or burns were excluded.
[4] Downgraded by 1 for serious imprecision: This subgroup of patients from the FEAST trial is severely underpowered to detect clinically important differences between the interventions.
[5] This study retrospectively compared mortality in children with septic shock who received >40 mls/kg during the first hour of treatment with those receiving <20 mls/kg.
[6] Downgraded by 1 for serious indirectness: This study was conducted in a pediatric intensive care unit in Pittsburgh USA, and over 80% had a severe pre-existing chronic disease such as malignancy.
[7] Downgraded by 1 for serious imprecision: The data were only presented as percentages and 95% CI could not be calculated. A P-value of <0.05 is stated by the authors.

[8] This study retrospectively examined the health records of children presenting with septic shock in the USA, requiring transport to a children hospital.

[9] Downgraded by 1 for risk of bias: This study compares ‘appropriate fluid therapy’ in line with ACCM/PALS guidelines with ‘inadequate fluid therapy’. The ‘appropriate therapy’ group includes those where fluid was given in line with ACCM/PALS guidelines AND those who recovered quickly irrespective of how much fluid was given.

[10] This study retrospectively examined health records of children with septic shock admitted to pediatric intensive care in Washington USA, and compares three groups receiving >40 mls/kg in the first hour, 20-40 mls/kg, and <20 mls/kg. Here we present > 40mls/ kg vs <40 mls/kg.

[11] Downgraded by 1 for serious imprecision: This trial is severely underpowered to confidently detect clinically important differences.

For peer review only

Table 6. Summary of findings and quality of evidence

Bolus fluids compared to maintenance fluids alone in children with severe febrile illness and any sign of impaired circulation (IC)

Patient or population: Children with severe febrile illness and some circulatory impairment[1]
Settings: Hospitals (East Africa, India)
Intervention: Bolus fluid resuscitation (40 mls/kg albumin or normal saline) followed by maintenance fluids (2.5 to 4.0 mls/kg)[2]
Comparison: Maintenance fluids alone (2.5 to 4.0 mls/kg)[3]

Outcomes	Study design	Illustrative comparative risks* (95% CI)		Relative effect (95% CI)	No of Participants (studies)	Quality of the evidence (GRADE)	Comments
		Assumed risk	Corresponding risk				
		**Maintenance fluids alone**	**Bolus followed by maintenance fluids**				
**Mortality** at 48 hours	RCT	**73 per 1000**	**106 per 1000** (82 to 135)	**RR 1.45** (1.13 to 1.86)	3,141 (1 study)	⊕⊕⊕⊕ **high**[1,2,3,4,5,6]	Maitland et al [12]
**Mortality** at 72 hours	RCT	**18 per 100**	**18 per 100** (9 to 36)	**RR 0.99** (0.49 to 1.98)	147 (1 study)	⊕⊕⊖⊖ **low**[7,8,9]	Santhanam et al [18]
**Mortality**	Prospective cohort	**14 per 100**	**12 per 100** (5 to 27)	**RR 0.84** (0.37 to 1.91)	187 (1 study)	⊕⊖⊖⊖ **very low**[10,11]	Carcillo et al [19]
**Mortality** at 4 weeks	RCT	**87 per 1000**	**121 per 1000** (97 to 152)	**RR 1.39** (1.11 to 1.74)	3,141 (1 study)	⊕⊕⊕⊖ **moderate**[4,5,6]	Maitland et al [12]
**Neurologic sequelae** at 4 weeks	RCT	**20 per 1000**	**21 per 1000** (12 to 35)	**RR 1.03** (0.61 to 1.75)	2,983 (1 study)	⊕⊕⊖⊖ **low**[4,5,12]	Maitland et al [12]
**Pulmonary edema, increased intracranial pressure**	RCT	**16 per 1000**	**24 per 1000** (14 to 41)	**RR 1.46** (0.85 to 2.53)	3,141 (1 study)	⊕⊕⊖⊖ **low**[4,5,12]	Maitland et al [12]
**Severe anaemia:** mortality at 48 hours	RCT	**90 per 1000**	**155 per 1000** (105 to 227)	**RR 1.71** (1.16 to 2.51)	987 (1 study)	⊕⊕⊕⊖ **moderate**[13]	Maitland et al [12]
**Non-severe anaemia:** mortality at 48 hours	RCT	**6 per 100**	**8 per 100** (6 to 12)	**RR 1.31** (0.93 to 1.84)	2,067 (1 study)	⊕⊕⊕⊖ **moderate**[14]	Maitland et al [12]

*The basis for the **assumed risk** (e.g. the median control group risk across studies) is provided in footnotes. The **corresponding risk** (and its 95% confidence interval) is based on the assumed risk in the comparison group and the **relative effect** of the intervention (and its 95% CI).
CI: Confidence interval; **RR:** Risk ratio

GRADE Working Group grades of evidence
High quality: Further research is very unlikely to change our confidence in the estimate of effect.
Moderate quality: Further research is likely to have an important impact on our confidence in the estimate of effect and may change the estimate.
Low quality: Further research is very likely to have an important impact on our confidence in the estimate of effect and is likely to change the estimate.
Very low quality: We are very uncertain about the estimate.

For peer review only - http://bmjopen.bmj.com/site/about/guidelines.xhtml

[1] The FEAST inclusion criteria were: One or more of: capillary refill >2 secs, lower limb temp gradient, weak pulse, tachycardia.
[2] The bolus was initially 20 mls/kg over 1 hour but this was increased to 40 mls/kg part-way through the trial following a preliminary analysis which showed increased mortality with the bolus.
[3] All children received antibiotics, maintenance fluids and supportive care according to standard guidelines.
[4] No serious risk of bias: Randomization and allocation concealment were adequate to reduce the risk of selection bias. Study staff were un-blinded to the intervention.
[5] No serious indirectness: Children with severe hypotension, severe malnutrition, gastroenteritis, or shock due to trauma surgery or burns were excluded.
[6] No serious imprecision: The result is clinically important and statistically significant.
[7] Children with septic shock in hospital in India were randomized to 20 to 40 mls/kg over 15 mins or 20 mls/kg over 20 mins up to a maximum of 60 mls/kg over an hour.
[8] Downgraded by 1 for serious indirectness: The comparison in this trial is not easily related to the comparison in FEAST.
[9] Downgraded by 1 for serious imprecision: The trial is underpowered to confidently rule out differences.
[10] This non-randomized study compares children with shock who received resuscitation in line with PALS guidelines early, and those that didn't. This subgroup excludes trauma cases, but includes cardiac, neurological, respiratory, sepsis and gastroenteritis as causes of shock.
[11] Downgraded by 1 for serious indirectness as the fluid volumes administered are not presented.
[12] Downgraded by 1 for serious imprecision: The 95% CI includes a relative risk of 1 (no effect) and appreciable benefit and harm.
[13] Severe anaemia, hemoglobin <5 g/dl: Downgraded by 1 for serious imprecision (95% CI includes appreciable harm) and few events (n=131 deaths).
[14] Non-severe anaemia, hemoglobin ≥5 g/dl: Downgraded by 1 for serious imprecision (95% CI includes 1 (no effect)) and few events (n=157 deaths).

For peer review only

PRISMA 2009 Checklist

Section/topic	#	Checklist item	Reported on page #
**TITLE**			
Title	1	Identify the report as a systematic review, meta-analysis, or both.	1
**ABSTRACT**			
Structured summary	2	Provide a structured summary including, as applicable: background; objectives; data sources; study eligibility criteria, participants, and interventions; study appraisal and synthesis methods; results; limitations; conclusions and implications of key findings; systematic review registration number.	2-3
**INTRODUCTION**			
Rationale	3	Describe the rationale for the review in the context of what is already known.	5
Objectives	4	Provide an explicit statement of questions being addressed with reference to participants, interventions, comparisons, outcomes, and study design (PICOS).	6
**METHODS**			
Protocol and registration	5	Indicate if a review protocol exists, if and where it can be accessed (e.g., Web address), and, if available, provide registration information including registration number.	No protocol available
Eligibility criteria	6	Specify study characteristics (e.g., PICOS, length of follow-up) and report characteristics (e.g., years considered, language, publication status) used as criteria for eligibility, giving rationale.	6
Information sources	7	Describe all information sources (e.g., databases with dates of coverage, contact with study authors to identify additional studies) in the search and date last searched.	6
Search	8	Present full electronic search strategy for at least one database, including any limits used, such that it could be repeated.	6
Study selection	9	State the process for selecting studies (i.e., screening, eligibility, included in systematic review, and, if applicable, included in the meta-analysis).	6
Data collection process	10	Describe method of data extraction from reports (e.g., piloted forms, independently, in duplicate) and any processes for obtaining and confirming data from investigators.	6-7
Data items	11	List and define all variables for which data were sought (e.g., PICOS, funding sources) and any assumptions and simplifications made.	7
Risk of bias in individual studies	12	Describe methods used for assessing risk of bias of individual studies (including specification of whether this was done at the study or outcome level), and how this information is to be used in any data synthesis.	7
Summary measures	13	State the principal summary measures (e.g., risk ratio, difference in means).	7

For peer review only

PRISMA 2009 Checklist

Synthesis of results	14	Describe the methods of handling data and combining results of studies, if done, including measures of consistency (e.g., I^2) for each meta-analysis.	7

Section/topic	#	Checklist item	Reported on page #
Risk of bias across studies	15	Specify any assessment of risk of bias that may affect the cumulative evidence (e.g., publication bias, selective reporting within studies).	7
Additional analyses	16	Describe methods of additional analyses (e.g., sensitivity or subgroup analyses, meta-regression), if done, indicating which were pre-specified.	7
**RESULTS**			
Study selection	17	Give numbers of studies screened, assessed for eligibility, and included in the review, with reasons for exclusions at each stage, ideally with a flow diagram.	7-8
Study characteristics	18	For each study, present characteristics for which data were extracted (e.g., study size, PICOS, follow-up period) and provide the citations.	8, table 1
Risk of bias within studies	19	Present data on risk of bias of each study and, if available, any outcome level assessment (see item 12).	8, tables 3-5
Results of individual studies	20	For all outcomes considered (benefits or harms), present, for each study: (a) simple summary data for each intervention group (b) effect estimates and confidence intervals, ideally with a forest plot.	8-10; tables 4 & 5
Synthesis of results	21	Present results of each meta-analysis done, including confidence intervals and measures of consistency.	8-10;
Risk of bias across studies	22	Present results of any assessment of risk of bias across studies (see Item 15).	8-10; tables 3-5
Additional analysis	23	Give results of additional analyses, if done (e.g., sensitivity or subgroup analyses, meta-regression [see Item 16]).	None.
**DISCUSSION**			
Summary of evidence	24	Summarize the main findings including the strength of evidence for each main outcome; consider their relevance to key groups (e.g., healthcare providers, users, and policy makers).	10-11
Limitations	25	Discuss limitations at study and outcome level (e.g., risk of bias), and at review-level (e.g., incomplete retrieval of identified research, reporting bias).	10-11
Conclusions	26	Provide a general interpretation of the results in the context of other evidence, and implications for future research.	10-11
**FUNDING**			

For peer review only

For peer review only - http://bmjopen.bmj.com/site/about/guidelines.xhtml

PRISMA 2009 Checklist

Funding	27	Describe sources of funding for the systematic review and other support (e.g., supply of data); role of funders for the systematic review.	12

From: Moher D, Liberati A, Tetzlaff J, Altman DG, The PRISMA Group (2009). Preferred Reporting Items for Systematic Reviews and Meta-Analyses: The PRISMA Statement. PLoS Med 6(6): e1000097. doi:10.1371/journal.pmed1000097

For more information, visit: **www.prisma-statement.org**.

For peer review only

For peer review only - http://bmjopen.bmj.com/site/about/guidelines.xhtml

BMJ Open

Immediate fluid management of children with severe febrile illness and signs of impaired circulation in low income settings: a contextualized systematic review

Journal:	*BMJ Open*
Manuscript ID:	bmjopen-2014-004934.R1
Article Type:	Research
Date Submitted by the Author:	09-Apr-2014
Complete List of Authors:	Opiyo, Newton ; Kenya Medical Research Institute/Wellcome Trust Research Programme, Health Services Unit<br>Molyneux, Elizabeth; College of Medicine and Queen Elizabeth Central Hospital, Department of Paediatrics<br>Sinclair, Dave; Liverpool School of Tropical Medicine, International Health Group<br>Garner, Paul; Liverpool School of Tropical Medicine, International Health Group<br>English, Mike; University of Oxford, Nuffield Department of Medicine and Department of Paediatrics
**Primary Subject Heading**:	Evidence based practice
Secondary Subject Heading:	Emergency medicine
Keywords:	Neonatal intensive & critical care < INTENSIVE & CRITICAL CARE, Paediatric intensive & critical care < INTENSIVE & CRITICAL CARE, PAEDIATRICS

SCHOLARONE™
Manuscripts

For peer review only

Immediate fluid management of children with severe febrile illness and signs of impaired circulation in low income settings: a contextualized systematic review

Newton Opiyo *Post-doctoral Researcher*[1*], Elizabeth Molyneux *Professor*[2], David Sinclair *Lecturer*[3], Paul Garner *Professor*[3], Mike English *Professor*[1, 4]

[1]Health Services Unit, KEMRI-Wellcome Trust Research Programme, Nairobi, 00100, Kenya; [2]Department of Paediatrics, College of Medicine and Queen Elizabeth Central Hospital, Blantyre, 3, Malawi; [3]International Health Group, Liverpool School of Tropical Medicine, Liverpool, L3 5QA, UK; [4]Nuffield Department of Medicine and Department of Paediatrics, University of Oxford, Oxford, OX3 7BN, UK

*** Corresponding author**

Newton Opiyo
KEMRI-Wellcome Trust Research Programme
P.O. Box 43640, Nairobi, 00100, Kenya
nopiyo@kemri-wellcome.org

**Word count**	Abstract	~~558~~518
	Main text	~~2,858~~2,864

For peer review only

For peer review only - http://bmjopen.bmj.com/site/about/guidelines.xhtml

Abstract

Objective

To evaluate the ~~safety and effectiveness~~effects of intravenous fluid bolus ~~(with either colloids or crystalloids) as part of immediate emergency care~~ compared to maintenance intravenous fluids alone as part of immediate emergency care in children with severe febrile illness and signs of impaired circulation in ~~from a~~ low income settings. ~~perspective.~~

Design

Systematic review of randomized controlled trials (RCTs), and observational studies, including retrospective analyses, that compare~~ing~~ fluid bolus regimens with maintenance fluids alone. ~~Our~~ The primary outcome measure was pre-discharge mortality.

Data sources and synthesis

We searched PubMed, The Cochrane Library (to January 2014), with complementary earlier searches on, Google Scholar and Clinical Trial Registries (~~up~~ to March 2013). ~~An updated MEDLINE search was done in January 2014.~~ As studies used different clinical signs to define impaired circulation we classified patients into those with signs of severely impaired circulation, or those with any signs of impaired circulation. ~~As the studies also used different fluid regimens, and incorporated different study designs, we did not pool data across studies.~~ The quality of evidence for each outcome was appraised using the Grading of Recommendations Assessment, Development and Evaluation (GRADE) approach. Findings are presented as risk ratios (RRs) with 95% confidence intervals (CIs).

Results

Six studies were included. Two were randomized controlled trials, one large trial (n=3141 children) from a low income country, and a smaller trial from a middle income country. ~~Two RCTs and four observational studies were included. One RCT from district hospitals in East Africa recruited 3141 children;~~ T~~t~~he remaining studies were from middle or high income settings, observational, and with few participants~~are smaller~~ (34 to 187 ~~participants~~children). ~~and all conducted in middle or high income settings with access to intensive care facilities.~~

Severely impaired circulation: The large RCT included a small subgroup ~~that met the criteria for~~with severely impaired circulation. ~~Mortality was higher~~There were more deaths in those receiving bolus fluids (20-40 mls/kg/hr, saline or albumin) compared to maintenance fluids (2.5-4 mls/kg/hr) (RR 2.40, 95% CI 0.84 to 6.88, P=0.054, 65 participants, *low quality evidence*). Three additional observational studies, all ~~in this group were~~ at high risk of confounding, ~~and~~ found mixed effects on mortality (*very low quality evidence*).

Any signs of impaired circulation: The RCT ~~from East Africa~~ included children with signs of both severely and non-severely impaired circulation. Overall, bolus fluids increased 48-hour mortality compared to maintenance fluids with an additional 3 deaths per 100 children treated (RR 1.45, 95% CI 1.13 to 1.86,

For peer review only

3141 participants, *high quality evidence*). In a second small RCT from India, no difference in 72-hour mortality was detected between children who received 20-40 mls/kg Ringers lactate over 15 minutes and those who received 20 mls over 20 minutes up to a maximum of 60 mls/kg over 1 hour (147 participants, *low quality evidence*).

In one additional observational study, resuscitation consistent with APLS guidelines, including fluids, was not associated with reduced mortality in the small sub-group with septic shock (*very low quality evidence*).

Signs of impaired circulation, but not severely impaired: Only the large RCT from East Africa allowed an analysis for children with some signs of impaired circulation who would not meet the criteria for severe impairment. Bolus fluids increased 48-hour mortality compared to maintenance alone (RR 1.36, 95% CI 1.05 to 1.76, *high quality evidence*).

Conclusion

Prior to the publication of the large multi-centre RCT from East Africa, the global evidence base for bolus fluid therapy in children with severe febrile illness and signs of impaired circulation was of very low quality., and poorly applicable to low resource settings. This large study provides robust evidence that in low resource income settings fluid boluses increase mortality in children with severe febrile illness and impaired circulation, and this increased risk is consistent across children with severe and less severe circulatory impairment.

Keywords: Febrile illness, fluid resuscitation, shock, children, systematic review

For peer review only - http://bmjopen.bmj.com/site/about/guidelines.xhtml

For peer review only

Strengths and limitations of this study

- Timely systematic review given the current uncertainty on optimal strategy for fluid resuscitation in children; review incorporates data on the largest randomised controlled trialRCT (FEAST trial) on of fluid therapy in paediatric emergency carechildren;

- Review includes a comprehensive search strategy, and uniquely uses the robust GRADE methodology to synthesize available evidenceall relevant comparative trials and observational data, and critically appraises the research evidence using GRADE methods;

- Review limitations include small number of studies and observational studies with small sample sizes.is limited because most studies are small and unreliable, with only one large RCT providing reliable data to guide policy.

For peer review only

Background

~~Severely ill children require health staff to~~Health staff must rapidly assess, resuscitate and treat ~~them to prevent death~~severely ill children to improve survival. Practical, simple to use protocols have been established to guide care.~~. in an often stressful, busy environment.~~ In North America, ~~and Europe,~~ the most commonly used are the Pediatric Advanced Life Support (PALS)[1] and in Europe, the European Paediatric Life Support (EPLS)[2] guidelines. ~~respectively.~~ In low income settings, ~~T~~the World Health Organization ~~have~~ has specific guidelines where there are no ~~for low income settings without~~ intensive care facilities, called the Emergency Triage Assessment and Treatment (ETAT) guidelines. ETAT guidance begins with initial triage to identify children who need urgent formal assessment such as altered consciousness or severe breathing or circulation problems. Immediate care should then follow an ABCD approach to assessment and action which prioritises care of the airway (A) first, followed by breathing (B) and circulation (C).

Septic shock

Severe febrile illness in children is often associated with signs of impaired circulation (also known as 'septic shock'). Current ETAT guidelines define shock as the presence of **three** clinical signs of poor peripheral perfusion: weak / absent peripheral pulse, prolonged capillary refilling >3 seconds, **and** cold hands and feet (typically with cold skin extending up the limb and termed 'a temperature gradient'). International guidance, including ETAT, typically recommends a rapid fluid bolus of 20 to 40 mls/kg intravenously once 'shock' is diagnosed.[3]

Why it is important to do this review

As early as 1999, authors were arguing that no robust data existed demonstrating that bolus fluid resuscitation improved clinical outcomes in septic patients.[4] Moreover, the results of observational studies suggested that large volumes of resuscitation fluids may be associated with increased morbidity in patients with sepsis[5], acute respiratory distress syndrome[6] or acute kidney injury.[7]

Subsequently, a large, multicentre, randomized controlled trial published in 2011, and conducted in East Africa found that bolus fluid resuscitation increased mortality compared to a maintenance fluid regimen in children with 'febrile illness' and impaired perfusion.[8] The results of this trial clashed with the long-standing belief that fluid bolus resuscitation was beneficial and caused considerable international debate.[9-12] To date, no clear guidance has emerged on how to incorporate these findings into recommendations for practice in high or low income settings.

Objective

To evaluate the ~~safety and effectiveness~~effects of fluid bolus (with either colloids or crystalloids) compared to maintenance fluids alone in children with severe febrile illness and signs of impaired circulation.

Methods

For peer review only - http://bmjopen.bmj.com/site/about/guidelines.xhtml

For peer review only

Criteria for considering studies for this review

Types of studies: Randomised controlled trials (RCTs), ~~quasi-experimental studies, controlled before and after studies and cohort studies.~~and observational studies, including retrospective analyses.

Types of participants: Children (aged ≤18 years) with clinical features suggesting impaired perfusion (including shock) due to presumed acute infectious illnesses or inflammatory state (sepsis) and excluding diarrhoeal illness (as defined by the studies).

Types of interventions: Intravenous fluid boluses (crystalloids or colloids) compared to no (or lower volume) fluid boluses or maintenance fluids.

Types of outcome measures: The primary outcome measure was pre-discharge mortality. Secondary outcomes were: mortality at any time up to 4 weeks and any adverse clinical events reported in the studies.

Data sources and search strategy

We searched PubMed, The Cochrane Library to January 2014, complemented by searches in Google Scholar and Clinical Trial Registries (ClinicalTrials.gov, Current Controlled Trials, WHO International Clinical Trials Registry Platform, metaRegister of Controlled Trials) to March 2013. ~~for eligible~~We sought eligible published, unpublished or in-progress articles. ~~The searches covered the period from the databases start dates to March 2013. An updated MEDLINE search was done in January 2014.~~ No date or language restrictions were used.

The searches were performed iteratively by combining MeSH (Medical Subject Headings) and free text terms relevant to the conditions (sepsis, septicemia, febrile illness, bacteremia, infection, meningitis, septic shock, hypovolemia), treatments (fluids, resuscitation, intravenous fluids, fluid therapy) and patient groups (neonates, infants, children, adolescents) of interest. No date or language restrictions were used.

In addition we searched the websites of relevant organizations (the International Sepsis Forum, the World Federation of Pediatric Intensive Care and Critical Care Societies) and key emergency / intensive care journals (Critical Care Medicine, Critical Care, Pediatric Emergency Medicine, Shock, Resuscitation, Intensive Care Medicine). Reference lists of related systematic reviews and primary studies were handsearched. We also sought additional papers by contacting authors of related reviews and selected studies.

Study selection

For peer review only

Two reviewers (NO and ME) independently screened the titles, abstracts and full texts of retrieved articles and applied the study eligibility criteria detailed above to select studies. Disagreements were resolved by discussion.

Data extraction

Data were extracted using a pre-designed form by one reviewer (NO) and checked by the other reviewers; disagreements were resolved by discussion. We extracted data on: study designs, settings, sample size, participants (diagnoses, age range), shock definitions, treatments and comparisons (types of fluids, timing, volumes and fluid rates), co-interventions and proportion of patients experiencing the events of interest in each treatment group.

Risk of bias in individual studies

Two reviewers (NO, DS) independently assessed the risk of bias in the included studies according to six criteria assessing the risk of selection bias (random sequence generation, allocation concealment, selection of two groups), reporting bias (blinding), and confounding (baseline characteristics, co-interventions). For each criteria, the study was classified as high risk of bias, low risk of bias or unclear risk of bias.

Assessment of quality of evidence

The quality of evidence for each of the efficacy and safety outcomes was assessed using the GRADE (Grading of Recommendation Assessment Development and Evaluation) approach.[13] Key quality elements assessed by GRADE include: risk of bias (study limitations), precision of treatment effects, consistency of results, directness (applicability) of evidence, and publication bias. The GRADE evidence profiles were prepared by one reviewer (NO) and verified independently by two reviewers (ME, DS). Discrepancies in the quality ratings were resolved by discussion.

Synthesis of results

We summarized results narratively due to significant differences in study designs, fluid protocols and patient risk profiles. In order to compare studies with similar populations we grouped studies by the severity of circulatory impairment at baseline, after an appraisal of the clinical signs used to define inclusion. We defined three groups (table 1):

1. *Severely impaired circulation (SIC):* Studies where inclusion criteria were similar to the ETAT guidance (shock defined as presence of all ~~three~~ four signs of impaired circulation).
2. *Impaired circulation (IC):* Studies where inclusion only required one or two of these signs.
3. *Impaired circulation but without severe impairment:* Studies where inclusion required one or two signs of impaired circulation and more severely ill patients were excluded.

For peer review only - http://bmjopen.bmj.com/site/about/guidelines.xhtml

For consistency all results are presented as risk ratios (RRs) with 95% confidence intervals (CIs) (where reported, odds ratios and percentage point differences were converted into risk ratios).

Results

Study selection process

The flow of studies through this review is summarized in Figure 1. Six studies fulfilled all our pre-specified eligibility criteria: two randomized controlled trials[8,14], two prospective cohort studies[15,16], and two retrospective record reviews.[17,18]

Study characteristics

The characteristics of the six included studies are summarized in ~~table 1~~Supplementary table 1. Study settings were varied: USA[15-17], Brazil[18], India[14] and East Africa (Kenya, Tanzania and Uganda).[8] Five studies[14-18] were conducted in settings where paediatric intensive care unit facilities including inotropic support, intubation and ventilation were available. The largest study[8], was conducted in typical resource-limited East African hospitals without these additional measures being available. The study sample sizes ranged from 34 to 3,141 patients.

The clinical definitions of severe febrile illness and circulatory impairment or 'shock' varied across studies (table 2). Fluid therapy protocols (volumes, timing, rates) were similarly varied between comparison study groups (~~table 1~~Supplementary table 1). In only one case, the work conducted in East Africa, was a clearly defined 'no bolus' group studied.[8]

Risk of bias

Both RCTs were considered to be of low risk of bias (table 3). The observational studies were all potentially confounded (none met all the risk of bias criteria; table 3). The following key criteria were only partially met or were unclear from the information provided: appropriate participant selection, study power, appropriate blinding/outcome assessments, and association of outcome and treatment.

~~Efficacy and safety o~~Outcomes

The results for the efficacy and safety outcomes, together with quality of evidence, are summarized in the following three distinct population risk groups.

(a) Children with severe febrile illness and signs of severely impaired circulation (SIC)

Four studies reported outcome data in children relevant to those with severely impaired circulation: one RCT[8], one prospective cohort[16], and two retrospective studies.[~~16~~17,18] Overall mortality in the patients reported in these analyses were: 42%, 51%, 29% and 47% respectively.

Trial data: The RCT[8] included children with milder forms of circulatory impairment but provided a sub-group analysis of 65 patients who fulfilled the ETAT criteria of 'shock'. In this subgroup a fluid bolus of 20 to 40 mls/kg (saline or albumin) over 1 hour was associated with a considerably higher risk of

For peer review only - http://bmjopen.bmj.com/site/about/guidelines.xhtml

mortality than maintenance fluids (2 to 4 mls/kg/hr) but this was of borderline statistical significance (RR 2.40, 95% CI, 0.84 to 6.88, P=0.054, *low quality evidence*; ~~table 4 and GRADE evidence profile 1~~Supplementary table 2 and Supplementary table 3).

Observational data: In Brazil a retrospective records review of 90 children admitted to a paediatric intensive care unit with sepsis and shock assessed the relationship between mortality and fluid resuscitation in the first hour.[18] Administration of 20 mls/kg of bolus resuscitation fluid (crystalloids, colloids) was associated with a significantly higher mortality than if 40 mls/kg or more was given (73% versus 33%, RR 0.45, 95% CI not estimable, P<0.05, *very low quality evidence*; ~~table 4 and GRADE evidence profile 1~~Supplementary table 2 and Supplementary table 3). Of particular note 80 of the 90 children studied had severe, pre-existing chronic disease such as malignancy.

A second retrospective records review from the USA, examined mortality in 91 children being resuscitated and referred by community physicians.[17] In this study 'appropriate fluid therapy' was reported to be strongly associated with improved survival (RR 0.20, 95% CI 0.05 to 0.78, *very low quality evidence*; ~~table 4 and GRADE evidence profile 1~~Supplementary table 2 and Supplementary table 3). However, the 'appropriate fluid therapy' group included those with fluids given as per guidelines and children in whom signs of shock resolved quickly irrespective of the volumes of fluid given. In fact, the median fluid volumes given to children who died (32.9 mls/kg) were higher than those given to survivors (20 mls/kg).

The third study, a prospective cohort from the USA, included 34 children admitted to the paediatric intensive care unit with microbiologically proven septic shock, and all were receiving inotropes and had a pulmonary catheter inserted.[16] Those receiving less than 20 mls/kg of fluids during the first hour of resuscitation (normal saline, Ringers lactate, 5% albumin) had significantly higher mortality than those given 20 to 40 mls/kg of fluids (RR 1.11, 95% CI 0.59 to 2.11, *very low quality evidence*) or more than 40 mls/kg (RR 0.19, 95% CI 0.03 to 1.30, *very low quality evidence*; ~~table 4 and GRADE evidence profile 1~~Supplementary table 2 and Supplementary table 3).

(b) Children with severe febrile illness and any sign of impaired circulation (IC)

Two randomized controlled trials[8,14] and one prospective cohort study[15] enrolled febrile children with clinical signs sufficiently similar to be included in the impaired circulation (IC) category. Overall mortality in the patients reported in these studies were 9.5%, 18% and 12.5% respectively.

Trial data: In the largest RCT from Africa 3,141 children were randomized to a fluid bolus (20 to 40 mls/kg albumin or normal saline over 1 hour) or maintenance fluids (2.5 to 4.0 mls/kg/hour).[8] Compared to maintenance fluids, the fluid bolus was associated with increased 48-hour mortality (RR 1.45, 95% CI 1.13 to 1.86, *high quality evidence*; ~~table 5 and GRADE evidence profile 2~~Supplementary table 4 and Supplementary table 5) and increased mortality at 4 weeks (RR 1.39, 95% CI 1.11 to 1.74, *moderate quality evidence*; ~~table 5 and GRADE evidence profile 2~~Supplementary table 4 and Supplementary table 5). There was no suggestion that albumin performed any differently than saline (albumin bolus vs. saline bolus, RR 1.0, 95% CI 0.78 to 1.29). There was no difference between the bolus and maintenance fluid groups in the risk of neurologic sequelae at 4 weeks (RR 1.03, 95% CI 0.61 to 1.75, *low quality evidence*;

For peer review only - http://bmjopen.bmj.com/site/about/guidelines.xhtml

~~table 5 and GRADE evidence profile 2~~Supplementary table 4 and Supplementary table 5) or the combined outcome of pulmonary edema or increased intracranial pressure (RR 1.46, 95% CI 0.85 to 2.53, *low quality evidence*; ~~table 5~~Supplementary table 4 and Supplementary table 5). A subgroup analysis suggested that the increased mortality was only statistically significant in children with severe anaemia (hemoglobin <5 g/dl) (RR 1.71, 95% CI 1.16 to 2.51, *moderate quality evidence*; ~~table 5 and GRADE evidence profile 2~~Supplementary table 4 and Supplementary table 5), but a subsequent analysis exploring the effect of anaemia when treated as a continuous variable found evidence of harm with fluid bolus across the full range of haemoglobin values.[19]

In the second RCT, 147 children in an Indian paediatric intensive care unit were randomized to receive Ringers lactate 20 to 40 mls/kg over 15 minutes or Ringers lactate 20 mls/kg over 20 minutes up to a maximum of 60 mls/kg over 1 hour.[14] There was no difference in 72-hour mortality between comparison groups (RR 0.99, 95% CI 0.49 to 1.98, *low quality evidence*; ~~table 5 and GRADE evidence profile 2~~Supplementary table 4 and Supplementary table 5).

Observational data: The prospective cohort study included 1,409 children but only 187 had septic shock and so were relevant to this review.[15] No difference in mortality was observed in this subgroup between those receiving resuscitation consistent with Pediatric Advanced Life Support (PALS)/Advanced Pediatric Life Support (APLS) recommendations (including rapid bolus 20 mls/kg of isotonic fluid, potentially repeated and use of inotropes) performed by community physicians compared to resuscitation not consistent with these recommendations (*very low quality evidence*; ~~table 5 and GRADE evidence profile 2~~Supplementary table 4 and Supplementary table 5). In addition, the actual fluid volumes administered and the relationship between fluid volumes given and outcomes is not presented. Furthermore, resuscitation episodes were classified as having been consistent with guidelines if signs of shock resolved early in the course of intervention, irrespective of actual fluid volumes given.

(c) Children with severe febrile illness and impaired circulation but not severely impaired circulation (IC but not SIC)

It was possible to derive outcome data for children with severe febrile illness and any sign of circulatory impairment (IC) but not severely impaired circulation (SIC) in one study[20]: 20 to 40 mls/kg bolus fluids (albumin, normal saline) provided over 1 hour, compared to 2.5 to 4.0 mls/kg/hr maintenance fluids, was associated in this large sub-group (n=3,076 children) with increased 48-hour mortality (RR 1.36, 95% CI 1.05 to 1.76, *high quality evidence*).

Discussion

This review was conducted to facilitate revision of national paediatric fluid management guidelines in Kenya and potentially neighbouring countries using ETAT guidance, but has direct policy implications for ~~rural~~ health care across Africa. ~~, and even for those involved in paediatric care in high resource settings.~~

The limited data available prior to the FEAST study demonstrate that the current recommendations for fluid bolus included in the ETAT guidelines were not supported by a strong scientific evidence base. In fact, the evidence was largely observational~~,~~ and ~~from intensive care settings not directly comparable to~~

For peer review only - http://bmjopen.bmj.com/site/about/guidelines.xhtml

~~typical resource-poor settings in Africa~~unreliable. Added to this, the studies were themselves flawed, with design aspects which potentially biased the result towards favouring high volume fluid resuscitation. Of particular note are two of the retrospective studies which classified all children who recovered quickly as having received 'adequate fluid therapy' irrespective of the fluid volume they received. The potential for bias in these studies resulting from possible exposure misclassification should be noted by paediatricians working in high resource settings.

The RCT published in 2011 provides by far the most direct assessment of fluid boluses in children with severe febrile illness in resource poor African settings that do not typically see Dengue Fever but where malaria may be common.[8] In these settings emergency management decisions must typically be made without accurate blood pressure reading or investigations such as pulse oximetry, blood gas analysis, haemoglobin or lactate measurement. The robust finding of increased mortality in the large group of children with an initial, clinical diagnosis of severe febrile illness and impaired circulation but not severely impaired circulation demonstrates that the clinical signs linked to this classification are, alone, not sufficient to identify children in whom fluid boluses may be beneficial. In fact this trial provides robust evidence that bolus intravenous fluids are harmful in such children.

Data from the small sub-group of children within the FEAST study who had all three clinical signs of severely impaired circulation, are the only randomized evidence on the risks and benefits of fluid boluses for this group. Mortality among those receiving bolus fluids was higher than those receiving maintenance fluids but the data are compatible with an effect ranging from a small potential benefit of bolus to very substantial harm, which raises severe doubt about the use of boluses even in this more severely ill group.

In an effort to accommodate the highly influential observational research on fluid use in paediatric emergency care we included data from studies traditionally excluded from systematic reviews of alternative therapies (such as the recent review by Ford and colleagues[21]). Although this presented challenges it is an advantage of the GRADE approach that the quality of evidence from such studies can be transparently appraised and, potentially therefore, contribute to informed decision making.

Conclusion

Prior to the publication of the large multicentre African RCT, the evidence in support of aggressive fluid therapy in children with septic shock was only of very low quality.~~, and poorly applicable to low resource settings.~~ The 2011 RCT provides robust evidence that in low resource settings fluid boluses, even in relatively small amounts, increase mortality in children with severe febrile illness and signs of impaired circulation. For children with signs of severely impaired circulation the evidence suggests harm but there is less certainty and further research is warranted.

For peer review only - http://bmjopen.bmj.com/site/about/guidelines.xhtml

Copyright

The Corresponding Author has the right to grant on behalf of all authors and does grant on behalf of all authors, an exclusive licence (or non exclusive for government employees) on a worldwide basis to the BMJ Publishing Group Ltd to permit this article (if accepted) to be published in BMJ editions and any other BMJPGL products and sublicences such use and exploit all subsidiary rights, as set out in our licence.

Competing interests

All authors have completed the Unified Competing Interest form atwww.icmje.org/coi_disclosure.pdf (available on request from the corresponding author) and declare: no support from any organisation for the submitted work; no financial relationships with any organisations that might have an interest in the submitted work in the previous three years, no other relationships or activities that could appear to have influenced the submitted work.

Elizabeth Molyneux worked with WHO to develop the initial ETAT training and supports the provision of ETAT training in Malawi and other countries. Mike English adapted ETAT when creating ETAT+ and supports the provision of ETAT+ training in Kenya and has facilitated use of ETAT+ in other countries.

Authors' contributions

NO conducted the searches. NO, DS and ME screened records for eligibility, extracted data, assessed study quality and analysed data. EM and PG reviewed selected studies. NO wrote the first draft of the manuscript. All the authors participated in the interpretation of results and writing of the full manuscript. NO and ME are the guarantors.

Ethical approval: Not required.

Funding

Newton Opiyo is funded by a post-doctoral training grant from the Consortium for National Health Research. Mike English is supported by a Wellcome Trust Senior Fellowship (#097170). Additional funds from a Wellcome Trust Strategic Award (#084538) and a Wellcome Trust core grant awarded to the KEMRI-Wellcome Trust Research Programme (#092654) made this work possible. The guideline development meeting was support in part by the Effective Health Care Research Consortium, which is funded by UKaid from the UK Government Department for International Development. The funding source had no role in study design, data collection, analysis, data interpretation, writing of the report or the decision to submit for publication. All authors carried out this research independently of the funding body.

For peer review only - http://bmjopen.bmj.com/site/about/guidelines.xhtml

Data sharing: No additional data available.

References

1. American Heart Association. 2005 American Heart Association (AHA) guidelines for cardiopulmonary resuscitation (CPR) and emergency cardiovascular care (ECC) of pediatric and neonatal patients: pediatric basic life support. Pediatrics 2006;117(5):e989-1004
2. Phillips B, Mackway-Jones K, Jewkes F. The European Resuscitation Council's paediatric life support course 'Advanced Paediatric Life Support'. Resuscitation 2000;47(3):329-334
3. Brierley J, Carcillo J, Choong K, *et al*. Clinical practice parameters for hemodynamic support of pediatric and neonatal septic shock: 2007 update from the American College of Critical Care Medicine. Crit Care Med 2009;37(2):666-88. Erratum in: Crit Care Med. 2009 Apr;37(4):1536.
4. Jardin F, Fourme T, Page B, *et al*. Persistent preload defect in severe sepsis despite fluid loading: A longitudinal echocardiographic study in patients with septic shock. Chest 1999;116(5):1354-9
5. Durairaj L, Schmidt G. Fluid therapy in resuscitated sepsis: less is more. Chest 2008;133(1):252-63
6. Wiedemann H. A perspective on the fluids and catheters treatment trial (FACTT). Fluid restriction is superior in acute lung injury and ARDS. Cleve Clin J Med 2008;75(1):42-8
7. Prowle J, Echeverri J, Ligabo E, *et al*. Fluid balance and acute kidney injury. Nat Rev Nephrol 2010;6(2):107-15
8. Maitland K, Kiguli S, Opoka R, *et al*. Mortality after fluid bolus in African children with severe infection. N Engl J Med 2011;364(26):2483-95
9. Brewster D. African fluid bolus study: implications for practice J Paediatr Child Health 2012;48(7):548-50
10. Duke T. What the African fluid-bolus trial means. Lancet 2011;378(9804):1685-7
11. Hilton A, Bellomo R. A critique of fluid bolus resuscitation in severe sepsis. Crit Care 2012;16(1):302
12. Southall D, Samuels M. Treating the wrong children with fluids will cause harm: response to 'mortality after fluid bolus in African children with severe infection'. Arch Dis Child 2011;96(10):905-6
13. GRADE Working Group. Available at: http://www.gradeworkinggroup.org/
14. Santhanam I, Sangareddi S, Venkataraman S, *et al.* A prospective randomized controlled study of two fluid regimens in the initial management of septic shock in the emergency department. Pediatr Emerg Care 2008;24(10):647-55
15. Carcillo J, Kuch B, Han Y, *et al*. Mortality and functional morbidity after use of PALS/APLS by community physicians. Pediatrics 2009;124(2):500-8
16. Carcillo J, Davis A, Zaritsky A. Role of early fluid resuscitation in pediatric septic shock. JAMA 1991;266(9):1242-5
17. Han Y, Carcillo J, Dragotta M, *et al*. Early reversal of pediatric-neonatal septic shock by community physicians is associated with improved outcome. Pediatrics 2003;112(4):793-9
18. Oliveira C, Nogueira dSF, Oliveira D, *et al*. Time- and fluid-sensitive resuscitation for hemodynamic support of children in septic shock: barriers to the implementation of the American College of Critical Care Medicine/Pediatric Advanced Life Support Guidelines in a pediatric intensive care unit in a developing world. Pediatr Emerg Care 2008;24(12):810-5
19. Maitland K, George E, Evans J, *et al*. Exploring mechanisms of excess mortality with early fluid resuscitation: insights from the FEAST trial. BMC Med 2013;11:68

For peer review only - http://bmjopen.bmj.com/site/about/guidelines.xhtml

20. Maitland K, Akech S, Russel E. Mortality after fluid bolus in African children with sepsis (Author's response). N Engl J Med 2011b;365(14):1351-1353

21. Ford N, Hargreaves S, Shanks L. Mortality after fluid bolus in children with shock due to sepsis or severe infection: a systematic review and meta-analysis. PLoS One 2012;7(8):e43953

Table 1. Severity of circulatory impairment classifications

Clinical group	Definition
Severely Impaired Circulation (SIC)	Children with severe febrile illness who have *all four* of the following features:<br>• AVPU<A<br>• Weak / absent peripheral pulse<br>• Prolonged capillary refilling >3 seconds<br>• Cold limb extremities (hands and feet) typically with cold skin extending up the limb (referred to as a temperature gradient)<br>These children typically also have secondary signs such as altered consciousness
Impaired Circulation (IC)	Children with severe febrile illness who may have *AVPU<A, or prostration or respiratory distress plus at least ONE* of the following features are included in this group:<br>• Weak peripheral pulse<br>• Capillary refilling >2 seconds<br>• Cold limb extremities with a temperature gradient<br>• Severe tachycardia (>180/min if aged 2-12m, >160/min if aged 1-4 years)
IC but without SIC	By exclusion a third clinical grouping can be defined, those with impaired circulation but without severe impairment

For peer review only - http://bmjopen.bmj.com/site/about/guidelines.xhtml

Table 2. Study inclusion criteria

	Study population entry criteria		'Shock' criteria													
Study	Age range	Severe illness		Blood pressure		Pulse rate		Capillary refill		Extremities		Peripheral pulse		Urine output		Mental status
**Maitland 2011**	60 days to 12 yrs	Severe febrile illness complicated by impaired consciousness (prostration or coma), respiratory distress (increased work of breathing) or both	**and**	—	**or**	Severe tachycardia†	**or**	≥ 3 secs	**or**	Lower limb temperature gradient	**or**	Weak radial pulse volume	—	—	—	—
**Oliveira 2008**	Median age: 36 to 47 months	Sepsis was defined using the Society of Critical Care Medicine Consensus Conference¶	**and**	< 5th percentile for age	**or**	—	**or**	< 1 sec or > 3 secs	**or**	Mottled/cool	**or**	Decreased	**or**	< 1 ml/kg/hr	**or**	Altered
**Han 2003**	1 to 131 months	Suspected infection as manifested by hyperthermia or hypothermia	**and**	< 5th percentile for age	**or**	—	**or**	> 3 secs	**or**	Mottled	**or**	Diminished	**or**	—		Decreased
**Carcillo 1991**	Median age 13.5 months (range 1 to 192 months)	Sepsis was diagnosed if the patient had a positive blood culture or if a pathologic organism from a tissue site was identified	**and**	< 2 SD below mean	**+ 3 of**	Tachycardia§§	**or**	—	**or**	Mottled/cool	**or**	Decreased	**or**	< 1 ml/kg/hr‡	—	—

For peer review only - http://bmjopen.bmj.com/site/about/guidelines.xhtml

**Santhanam 2008**	1 to 12 months	Septic shock was defined using the Sepsis Consensus Conference criteria§	**and**	—	**or**	Tachycardia	**+ 1 of**	> 2 secs	**or**	Mottled/cool	**or**	Decreased	**or**	Decreased	**or**	Altered alertness
**Carcillo 2009**	Newborn to 18 years	Unclear from the information provided††	**and**	< 5th percentile for age	**or**	Tachycardia	**or**	> 3 secs	**or**	Mottled	**or**	—	**or**	—	**or**	Altered

† >180 beats per minute in children younger than 12 months of age, >160 beats per minute in children 1 to 5 years of age, or 140 beats per minute in children older than 5 years of age;
§ Levy MM, Fink MP, Marshall JC, et al. 2001 SCCM/ESICM/ACCP/ ATS/ SIS International Sepsis Definitions Conference. Crit Care Med. 2003;31:1250-1256;
¶ Bone RC, Balk RA, Cerra FB, et al. Definitions for sepsis and organ failure and guidelines for the use of innovative therapies in sepsis. The ACCP/SCCM Consensus Conference Committee. American College of Chest Physicians/Society of Critical Care Medicine. Chest. 1992; 101(6): 1644-1655;
SD - Standard deviation;
§§ Heart rate >180 beats per minute for patients less than 5 years of age; and >160 beats per minute for patients at least 5 years of age;
‡Or less than 20 ml/hr in children weighing more than 20 kg

For peer review only - http://bmjopen.bmj.com/site/about/guidelines.xhtml

Table 3. Quality assessment of included studies

Study	Study design	Selection bias			Reporting bias	Confounding	
		Random sequence generation	Allocation concealment	Selection of two groups	Blinding	Baseline characteristics	Co-interventions
Maitland 2011 [8]	Randomized controlled trial	Low risk	Low risk	Low risk	Low risk	Low risk	Low risk
Santhanam 2008 [14]	Randomized controlled trial	Low risk	Low risk	Low risk	Low risk	Low risk	Low risk
Carcillo 2009 [15]	Prospective cohort	N/A	N/A	High risk[1]	N/A	High risk[1]	Unclear risk
Oliveira 2008 [18]	Retrospective records review	N/A	N/A	High risk[2]	N/A	High risk[2]	Unclear risk
Han 2003 [17]	Retrospective records review	N/A	N/A	High risk[3]	N/A	High Risk[3]	Unclear risk
Carcillo 1991 [16]	Prospective cohort	N/A	N/A	Low risk	N/A	Unclear risk[4]	Unclear risk

[1] Compared those who received recommended APLS/PALS treatment with those who didn't. 'Received recommended APLS/PALS fluid therapy' was defined as those who recovered regardless of fluid therapy plus those who didn't recover but received >20 mg/kg of fluids. Children who didn't receive recommended APLS/PALS treatment were significantly younger and had significantly longer capillary refill times, lower blood pressure, and higher oxygen requirements;
[2] Compared survivors and non-survivors. The survivors were more likely to have had higher fluid volumes and were also significantly younger;
[3] Compared survivors and non-survivors. 'Appropriate fluid therapy' group includes those where fluid was given in line with ACCM/PALS guidelines AND those who recovered quickly irrespective of how much fluid was given. Non-survivors had significantly higher PRISM scores at baseline (PRISM assesses the risk of mortality);
[4] Compared those who received three different fluid regimens. Adequate baseline characteristics were not presented.

For peer review only - http://bmjopen.bmj.com/site/about/guidelines.xhtml

Supplementary table 1. Characteristics of included studies

Study	Study design	Setting	Number of children	Intervention	Control	Co-interventions
Maitland 2011 [8]	RCT	Kenya Tanzania Uganda	3,141	20 to 40 mls/kg of 5% albumin or 0.9% saline over the first hour, followed by maintenance fluids	Intravenous maintenance fluids alone (2.5 to 4.0 mls/kg/hr)	Antibiotics, antimalarials, antipyretics, anticonvulant drugs, hypoglycemia treatment and blood transfusion
Santhanam 2008 [14]	RCT	India	147	20 to 40 mls/kg of Ringers lactate over 15 minutes followed by dopamine if therapeutic goal not achieved after 15 minutes	20 mls/kg over 20 minutes up to 60 mls/kg over 1 hour followed by dopamine if therapeutic goal not achieved at 1 hour	Antibiotics, anticonvulsant drugs, intubation, ventilation support, treatment of hypoglycemia, hypocalcemia and asthma
Carcillo 2009 [15]	Prospective cohort	USA	187 (a subgroup)	Received PALS/APLS - recommended resuscitation (20 to 40 mls/kg) or recovered quickly	Did not receive PALS/APLS - recommended resuscitation	Inotropic therapy
Han 2003 [17]	Retrospective records review	USA	91	Received PALS/APLS recommended resuscitation (>60 mls/kg normal saline) or recovered	Did not receive PALS/APLS recommended resuscitation	Antibiotics, inotropic/vasopressor support and hydrocortisone therapy
Carcillo 1991 [16]	Prospective cohort	USA	34	> 40 mls/kg total fluids (normal saline, Ringers lactate, 5% albumin or blood products)	<20 mls/kg total fluids or 20 to 40 mls/kg total fluids	Antibiotics, vasopressor and/or inotropic support and assisted ventilation
Oliveira 2008 [18]	Retrospective records review	Brazil	90	>40 mls/kg in the first hour (colloids, crystalloids, or packed red blood cells)	<20 mls/kg in the first hour	Antibiotics and inotropic support

For peer review only

For peer review only - http://bmjopen.bmj.com/site/about/guidelines.xhtml

Supplementary table 1. Characteristics of included studies (continued)

Study	Study design	Definition of severe febrile illness	Definition of circulatory impairment	Our classification	Overall mortality
Maitland 2011 [8]	Randomized controlled trial	Febrile illness with impaired consciousness and/or respiratory distress	One or more of: capillary refilling time ≥3 secs, severe tachycardia, temperature gradient or weak pulse	Some circulatory impairment (IC)<br><br>(Subgroup of severely impaired circulation (SIC))	9.5%<br><br>(42%)
Santhanam 2008 [14]	Randomized controlled trial	Children with septic shock	Tachycardia with signs of decreased perfusion including: decreased peripheral pulses compared with central pulses, altered alertness, flash capillary refill or capillary refill >2 secs, mottled or cool extremities, or decreased urine output	Some circulatory impairment (IC)	18%
Carcillo 2009 [15]	Prospective cohort	Infants and children with trauma and non-trauma diagnoses (including septic shock)	Prolonged capillary refill time (>3 seconds or mottled extremities) and/or hypotension (systolic blood pressure less than the fifth percentile for age according to PALS/APLS criteria)	Some circulatory impairment (IC)	12.5%
Han 2003 [17]	Retrospective records review	Infants and children with septic shock	Decreased perfusion, including decreased mental status, prolonged capillary refill time (>3 seconds), diminished peripheral pulses, or mottled extremities and hypotension (systolic blood pressure less than the fifth percentile for age)	Severe circulatory impairment (SIC)	29%
Carcillo 1991 [16]	Prospective cohort	Children with septic shock	Blood pressure <2 SDs below the mean for age, combined with three of the following four criteria for decreased perfusion: decreased peripheral pulses; mottled or cool extremities; tachycardia (heart rate >180 bpm for patients <5 years of age; and >160 bpm for patients ≥5 years of age); or urine output <1 mls/kg per hour (or <20 mls/hr in children weighing >20 kg)	Severe circulatory impairment (SIC)	51%
Oliveira 2008 [18]	Retrospective records review	Children with sepsis and septic shock	Decreased perfusion (decreased peripheral pulses, mottled or cool extremities, capillary refill time <1 second or >3 seconds), hypotension (systolic blood	Severe circulatory impairment (SIC)	47%

For peer review only - http://bmjopen.bmj.com/site/about/guidelines.xhtml

	pressure less than the fifth percentile for age, using the PALS formula), altered mental status, or oliguria (<1 mls/kg per hour)

bpm: Beats per minute; PALS: Pediatric Advanced Life Support; APLS: Advanced Pediatric Life Support; SD: Standard deviation;
IC: Severe febrile illness and any sign of impaired circulation;
SIC: Severe febrile illness and signs of severely impaired circulation;
IC but not SIC: Severe febrile illness and impaired circulation but not severely impaired circulation

For peer review only

For peer review only - http://bmjopen.bmj.com/site/about/guidelines.xhtml

Supplementary table 2. Summary of findings and quality of evidence

Bolus fluids compared to maintenance fluids alone in children with severe febrile illness and severely impaired circulation (SIC)

Patient or population: Children with severe febrile illness and severely impaired circulation
Settings: Hospitals (East Africa, USA, Brazil)
Intervention: Bolus fluid resuscitation (albumin or normal saline) followed by maintenance fluids
Comparison: Maintenance fluids alone[1]

**Outcomes**	**Study design**	Assumed risk	Corresponding risk (95% CI)	**Relative effect (95% CI)**	**No of Participants (studies)**	**Quality of the evidence (GRADE)**	**Comments**
		**Maintenance fluids alone**	**Bolus followed by maintenance fluids**				
**Mortality**	RCT	**20 per 100**	**48 per 100** (17 to 138)	**RR 2.40** (0.84 to 6.88)	65 (1 study)	⊕⊕⊖⊖ **Low**[2,3,4,5]	Maitland 2011 [8]
	Prospective cohort	**60 per 100**	**11 per 100** (2 to 73)	**RR 0.19** (0.03 to 1.21)	34 (1 study)	⊕⊖⊖⊖ **Very low**[6,7]	Carcillo 1991 [16]
	Retrospective cohort	**73 per 100**	**33 per 100** (NA)	**RR 0.45** (NA)	90 (1 study)	⊕⊖⊖⊖ **Very low**[8,9,10]	Oliveira 2008 [18]
	Retrospective cohort	**38 per 100**	**8 per 100** (2 to 30)	**RR 0.20** (0.05 to 0.78)	91 (1 study)	⊕⊖⊖⊖ **Very low**[11,12]	Han 2003 [17]

The **corresponding risk** (and its 95% confidence interval) is based on the assumed risk in the comparison group and the **relative effect** of the intervention (and its 95% CI).
CI: Confidence interval; **RR:** Risk ratio

GRADE Working Group grades of evidence
High quality: We are very confident that the true effect lies close to that of the estimate of the effect.
Moderate quality: We are moderately confident in the effect estimate: The true effect is likely to be close to the estimate of the effect, but there is a possibility that it is substantially different.
Low quality: Our confidence in the effect estimate is limited: The true effect may be substantially different from the estimate of the effect.
Very low quality: We have very little confidence in the effect estimate: The true effect is likely to be substantially different from the estimate of effect.

[1] All children received antibiotics, maintenance fluids and supportive care according to standard guidelines;
[2] This data represents a sub-group of patients from the large FEAST trial in East Africa [8]. These 65 patients had signs of a febrile illness and fulfilled the ETAT definition of shock;
[3] No serious risk of bias: Randomization and allocation concealment were adequate to reduce the risk of selection bias. Study staff were unblinded to the intervention;
[4] Downgraded by 1 for serious indirectness: Children with severe hypotension were excluded;
[5] Downgraded by 1 for serious imprecision: This subgroup of patients from the FEAST trial is severely underpowered to detect clinically important differences between the interventions;
[6] This study retrospectively examined health records of children with septic shock admitted to pediatric intensive care in Washington USA, and compares three groups receiving >40 mls/kg in the first hour, 20-40 mls/kg, and <20 mls/kg. Here we present > 40mls/ kg vs <40 mls/kg;
[7] Downgraded by 1 for serious imprecision: This trial is severely underpowered to confidently detect clinically important differences;
[8] This study retrospectively compared mortality in children with septic shock who received >40 mls/kg during the first hour of treatment with those receiving <20 mls/kg;
[9] Downgraded by 1 for serious indirectness: This study was conducted in a pediatric intensive care unit in Sao Paulo, Brazil, and over 80% had a severe pre-existing chronic disease such as malignancy;
[10] Downgraded by 1 for serious imprecision: The data were only presented as percentages and 95% CI could not be calculated. A P-value of <0.05 is stated by the authors;

For peer review only - http://bmjopen.bmj.com/site/about/guidelines.xhtml

[11] This study retrospectively examined the health records of children presenting with septic shock in the USA, requiring transport to a children hospital;
[12] Downgraded by 1 for risk of bias: This study compares 'appropriate fluid therapy' in line with ACCM/PALS guidelines with 'inadequate fluid therapy'. The 'appropriate therapy' group includes those where fluid was given in line with ACCM/PALS guidelines AND those who recovered quickly irrespective of how much fluid was given.

For peer review only

Supplementary table 3. Bolus fluids compared to maintenance fluids alone in children with severe febrile illness and severely impaired circulation (SIC)

Quality assessment						Summary of findings				
						Number of patients		Effect size		
**Study design**	**Risk of bias**	**Inconsistency**	**Indirectness**	**Imprecision**	**Publication bias**	**No bolus**	**Bolus**	**Relative risk (95% CI)**	**Absolute effect**	**Quality**
**Outcome: Mortality**										
Randomised control trial‡ [8]	No serious risk of bias[1]	No serious inconsistency	Serious[2]	Serious[3]	Undetected	3/15 (20%)	24/50 (48%)	RR 2.40 (0.84 to 6.88)	28 more per 100 (from 3 fewer to 100 more)	⊕⊕⊝⊝ Low
Prospective cohort study† [16]	Serious[4]	No serious inconsistency	No serious indirectness	Serious[5]	Undetected	15/25 (60%)	1/9 (11%)	RR 0.19 (0.03 to 1.21)	49 fewer per 100 (from 58 fewer to 13 more)	⊕⊝⊝⊝ Very low
Retrospective cohort study¶ [18]	Serious[6]	No serious inconsistency	Serious[7]	Serious[8]	Undetected	73%††	33%††	RR 0.45 95% CI not estimatable	Not estimatable	⊕⊝⊝⊝ Very low
Retrospective cohort study‡‡ [17]	Serious[9]	No serious inconsistency	No serious indirectness	Serious[10]	Undetected	24/64 (37.5%)	2/27 (7.4%)	RR 0.20 (0.05 to 0.78)	30 fewer per 100 (from 8 fewer to 36 fewer)	⊕⊝⊝⊝ Very low

CI: Confidence interval;

‡This data represents a sub-group of patients from the large FEAST trial in East Africa [8]. These 65 patients had signs of a febrile illness and fulfilled the ETAT definition of shock. The intervention comprised bolus fluid resuscitation (40 mls/kg albumin or normal saline) followed by maintenance fluids (2.5 to 4.0 mls/kg). The control (no bolus) group received maintenance fluids alone (2.5 to 4.0 mls/kg);

[1]No serious risk of bias: Randomization and allocation concealment were adequate to reduce the risk of selection bias. Study staff were unblinded to the intervention;

[2]Downgraded by 1 for serious indirectness: Children with severe hypotension were excluded;

[3]Downgraded by 1 for serious imprecision: This subgroup of patients from the FEAST trial is severely underpowered to detect clinically important differences between the interventions;

†This study retrospectively examined health records of children with septic shock admitted to pediatric intensive care in Washington USA, and compares three groups receiving >40 mls/kg in the first hour, 20-40 mls/kg, and <20 mls/kg. Here we present > 40mls/ kg vs <40 mls/kg;

[4]Adequate baseline characteristics were not presented;

[5]Downgraded by 1 for serious imprecision: This trial is severely underpowered to confidently detect clinically important differences;

¶This study retrospectively compared mortality in children with septic shock who received >40 mls/kg during the first hour of treatment with those receiving <20 mls/kg;

[6]Downgraded by 1 for risk of bias: The study compared survivors and non-survivors. The survivors were more likely to have had higher fluid volumes and were also significantly younger;

[7]Downgraded by 1 for serious indirectness: This study was conducted in a pediatric intensive care unit in Sao Paulo, Brazil, and over 80% had a severe pre-existing chronic disease such as malignancy;

[8]Downgraded by 1 for serious imprecision: The data were only presented as percentages and 95% CI could not be calculated. A P-value of <0.05 is stated by the authors;

†† A total of 45 children with septic shock died – however the number of deaths in the treatment groups was unclear (reported as 33%, >40 mls/kg and 73%, <20 mls/kg during the first hour of treatment);

‡‡ This study retrospectively examined the health records of children presenting with septic shock in the USA, requiring transport to a children hospital;

[9]Downgraded by 1 for risk of bias: This study compares 'appropriate fluid therapy' in line with ACCM/PALS guidelines with 'inadequate fluid therapy'. The 'appropriate therapy' group includes those where fluid was given in line with ACCM/PALS guidelines AND those who recovered quickly irrespective of how much fluid was given;

[10]Downgraded by 1 for serious imprecision: Small sample size (N=91 patients). Small number of events (N=26 deaths).

For peer review only - http://bmjopen.bmj.com/site/about/guidelines.xhtml

Supplementary table 4. Summary of findings and quality of evidence

Bolus fluids compared to maintenance fluids alone in children with severe febrile illness and any sign of impaired circulation (IC)

Patient or population: Children with severe febrile illness and some circulatory impairment
Settings: Hospitals (East Africa, India, USA)
Intervention: Bolus fluid resuscitation (albumin or normal saline) followed by maintenance fluids
Comparison: Maintenance fluids alone[1]

Outcomes	Study design	Assumed risk	Corresponding risk (95% CI)	Relative effect (95% CI)	No of Participants (studies)	Quality of the evidence (GRADE)	Comments
		Maintenance fluids alone	Bolus followed by maintenance fluids				
**Mortality** at 48 hours	RCT	**73 per 1000**	**106 per 1000** (82 to 135)	**RR 1.45** (1.13 to 1.86)	3,141 (1 study)	⊕⊕⊕⊕ **High**[2,3,4,5,6]	Maitland 2011 [8]
**Mortality** at 72 hours	RCT	**18 per 100**	**18 per 100** (9 to 35)	**RR 0.99** (0.49 to 1.98)	147 (1 study)	⊕⊕⊝⊝ **Low**[7,8,9]	Santhanam 2008 [14]
**Mortality**	Prospective cohort	**14 per 100**	**12 per 100** (5 to 27)	**RR 0.84** (0.37 to 1.91)	187 (1 study)	⊕⊝⊝⊝ **Very low**[10,11]	Carcillo 2009 [15]
**Mortality** at 4 weeks	RCT	**87 per 1000**	**121 per 1000** (97 to 152)	**RR 1.39** (1.11 to 1.74)	3,141 (1 study)	⊕⊕⊕⊕ **High**[4,5,6]	Maitland 2011 [8]
**Neurologic sequelae** at 4 weeks	RCT	**20 per 1000**	**21 per 1000** (12 to 35)	**RR 1.03** (0.61 to 1.75)	2,983 (1 study)	⊕⊕⊕⊝ **Moderate**[4,5,12]	Maitland 2011 [8]
**Pulmonary edema, increased intracranial pressure**	RCT	**16 per 1000**	**24 per 1000** (14 to 41)	**RR 1.46** (0.85 to 2.53)	3,141 (1 study)	⊕⊕⊕⊝ **Moderate**[4,5,12]	Maitland 2011 [8]
**Severe anaemia:** mortality at 48 hours	RCT	**90 per 1000**	**155 per 1000** (105 to 227)	**RR 1.71** (1.16 to 2.51)	987 (1 study)	⊕⊕⊕⊝ **Moderate**[13]	Maitland 2011 [8]
**Non-severe anaemia:** mortality at 48 hours	RCT	**6 per 100**	**8 per 100** (6 to 12)	**RR 1.31** (0.93 to 1.84)	2,067 (1 study)	⊕⊕⊕⊝ **Moderate**[14]	Maitland 2011 [8]

The **corresponding risk** (and its 95% confidence interval) is based on the assumed risk in the comparison group and the **relative effect** of the intervention (and its 95% CI).
CI: Confidence interval; **RR:** Risk ratio

GRADE Working Group grades of evidence
High quality: We are very confident that the true effect lies close to that of the estimate of the effect.
Moderate quality: We are moderately confident in the effect estimate: The true effect is likely to be close to the estimate of the effect, but there is a possibility that it is substantially different.
Low quality: Our confidence in the effect estimate is limited: The true effect may be substantially different from the estimate of the effect.
Very low quality: We have very little confidence in the effect estimate: The true effect is likely to be substantially different from the estimate of effect.

[1] All children received antibiotics, maintenance fluids and supportive care according to standard guidelines;
[2] The FEAST inclusion criteria were: One or more of: capillary refill >2 secs, lower limb temp gradient, weak pulse, tachycardia;

For peer review only - http://bmjopen.bmj.com/site/about/guidelines.xhtml

[3]The bolus was initially 20 mls/kg over 1 hour but this was increased to 40 mls/kg part-way through the trial following a preliminary analysis which showed increased mortality with the bolus;

[4] No serious risk of bias: Randomization and allocation concealment were adequate to reduce the risk of selection bias. Study staff were unblinded to the intervention;

[5] No serious indirectness: Children with severe hypotension, severe malnutrition, gastroenteritis, or shock due to trauma surgery or burns were excluded;

[6] No serious imprecision: The result is clinically important and statistically significant;

[7]Children with septic shock in hospital in India were randomized to 20 to 40 mls/kg over 15 mins or 20 mls/kg over 20 mins up to a maximum of 60 mls/kg over an hour;

[8] Downgraded by 1 for serious indirectness: The comparison in this trial is not easily related to the comparison in FEAST;

[9] Downgraded by 1 for serious imprecision: The trial is underpowered to confidently rule out differences;

[10] This non-randomized study compares children with shock who received resuscitation in line with PALS guidelines early, and those that didn't. This subgroup excludes trauma cases, but includes cardiac, neurological, respiratory, sepsis and gastroenteritis as causes of shock;

[11] Downgraded by 1 for serious indirectness as the fluid volumes administered are not presented;

[12] Downgraded by 1 for serious imprecision: The 95% CI includes a relative risk of 1 (no effect) and appreciable benefit and harm;

[13] Severe anaemia, hemoglobin <5 g/dl: Downgraded by 1 for serious imprecision (95% CI includes appreciable harm) and few events (n=131 deaths);

[14] Non-severe anaemia, hemoglobin ≥5 g/dl: Downgraded by 1 for serious imprecision (95% CI includes 1 (no effect)) and few events (n=157 deaths).

For peer review only

Supplementary table 5. Bolus fluids compared to maintenance fluids alone in children with severe febrile illness and any sign of impaired circulation (IC)

Quality assessment						Summary of findings				
						Number of patients		Effect size		
**Outcomes Study design**	**Risk of bias**	**Inconsistency**	**Indirectness**	**Imprecision**	**Publication bias**	**No bolus**	**Bolus**	**Relative risk (95% CI)**	**Absolute effect**	**Quality**
Mortality at 48 hours, RCT‡ [8]	No serious risk of bias[1]	No serious inconsistency	No serious indirectness[2]	No serious imprecision[3]	Undetected	76/1044 (7.3%)	221/2097 (10.5%)	RR 1.45 (1.13 to 1.86)	33 more per 1000 (from 9 more to 63 more)	⊕⊕⊕⊕ High
Mortality at 72 hours, RCT† [14]	No serious risk of bias[4]	No serious inconsistency	Serious[5]	Serious[6]	Undetected	13/73 (17.8%)	13/74 (17.6%)	RR 0.99 (0.49 to 1.98)	0 fewer per 100 (from 9 fewer to 17 more)	⊕⊕⊖⊖ Low
Mortality Prospective cohort ¶ [15]	Serious[7]	No serious inconsistency	Serious[8]	Serious[9]	Undetected	18/128 (14.1%)	7/59 (11.9%)	RR 0.84 (0.37 to 1.91)	2 fewer per 100 (from 9 fewer to 13 more)	⊕⊖⊖⊖ Very low
Mortality at 4 weeks, RCT [8]	No serious risk of bias[1]	No serious inconsistency	No serious indirectness[2]	No serious imprecision[3]	Undetected	91/1044 (8.7%)	254/2097 (12.1%)	RR 1.39 (1.11 to 1.74)	34 more per 1000 (from 10 more to 65 more)	⊕⊕⊕⊕ High
Neurologic sequelae at 4 weeks, RCT [8]	No serious risk of bias[1]	No serious inconsistency	No serious indirectness[2]	Serious[10]	Undetected	20/997 (2.0%)	41/1986 (2.1%)	RR 1.03 (0.61 to 1.75)	1 more per 1000 (from 8 fewer to 15 more)	⊕⊕⊖⊖ Moderate
Pulmonary edema, increased intracranial pressure, RCT [8]	No serious risk of bias[1]	No serious inconsistency	No serious indirectness[2]	Serious[10]	Undetected	17/1044 (1.6%)	50/2097 (2.4%)	RR 1.46 (0.85 to 2.53)	7 more per 1000 (from 2 fewer to 25 more)	⊕⊕⊖⊖ Moderate
Severe anaemia: mortality at 48 hours, RCT [8]	No serious risk of bias[1]	No serious inconsistency	No serious indirectness[2]	Serious[11]	Undetected	30/332 (9%)	101/655 (15.4%)	RR 1.71 (1.16 to 2.51)	64 more per 1000 (from 14 more to 136 more)	⊕⊕⊖⊖ Moderate
Non-severe anaemia: mortality at 48 hours, RCT [8]	No serious risk of bias[1]	No serious inconsistency	No serious indirectness[2]	Serious[12]	Undetected	43/683 (6.3%)	114/1384 (8.2%)	RR 1.31 (0.93 to 1.84)	20 more per 1000 (from 4 fewer to 53 more)	⊕⊕⊖⊖ Moderate

For peer review only - http://bmjopen.bmj.com/site/about/guidelines.xhtml

RCT: Randomised controlled trial;
‡Children were randomized to a fluid bolus (20 to 40 mls/kg albumin or normal saline over 1 hour) or maintenance fluids (2.5 to 4.0 mls/kg/hour);
[1]No serious risk of bias: Randomization and allocation concealment were adequate to reduce the risk of selection bias. Study staff were unblinded to the intervention;
[2]No serious indirectness: Children with severe hypotension, severe malnutrition, gastroenteritis, or shock due to trauma surgery or burns were excluded;
[3]No serious imprecision: The result is clinically important and statistically significant;
†Children with septic shock in hospital in India were randomized to 20 to 40 mls/kg over 15 mins or 20 mls/kg over 20 mins up to a maximum of 60 mls/kg over an hour;
[4]Low risk of selection bias (random sequence generation and allocation concealment adequate); low risk of reporting bias;
[5]Downgraded by 1 for serious indirectness: The comparison in this trial is not easily related to the comparison in FEAST trial [8];
[6]Downgraded by 1 for serious imprecision: The trial is underpowered to confidently rule out differences;
¶ This non-randomized study compares children with shock who received resuscitation in line with PALS guidelines early, and those that didn't. This subgroup excludes trauma cases, but includes cardiac, neurological, respiratory, sepsis and gastroenteritis as causes of shock;
[7]High risk for selection bias and confounding: 'Received recommended APLS/PALS fluid therapy' was defined as those who recovered regardless of fluid therapy plus those who didn't recover but received >20 mg/kg of fluids. Children who didn't receive recommended APLS/PALS treatment were significantly younger and had significantly longer capillary refill times, lower blood pressure, and higher oxygen requirements;
[8]Downgraded by 1 for serious indirectness as the fluid volumes administered are not presented;
[9]Downgraded by 1 for serious imprecision: Small sample size (N=187 patients) and small number of events (N=25 deaths due to sepsis);
[10]Downgraded by 1 for serious imprecision: The 95% CI includes a relative risk of 1 (no effect) and appreciable benefit and harm;
[11]Severe anaemia, hemoglobin <5 g/dl: Downgraded by 1 for serious imprecision (95% CI includes appreciable harm) and few events (n=131 deaths);
[12]Non-severe anaemia, hemoglobin ≥5 g/dl: Downgraded by 1 for serious imprecision (95% CI includes 1 (no effect)) and few events (n=157 deaths).

review only

For peer review only - http://bmjopen.bmj.com/site/about/guidelines.xhtml

Figure Legends

Figure 1. Results of literature search and studies selected

Table Legends

Table 1. ~~Characteristics of included studies~~Severity of circulatory impairment classification

Table 2. Study inclusion criteria

Table 3. Study quality assessment

Supplementary table 1. Characteristics of included studies

Supplementary table 2. SIC Summary of findings and quality of evidence

Supplementary table 3. Bolus fluids compared to maintenance fluids alone in children with severe febrile illness and severely impaired circulation (SIC)

Supplementary table 4. IC Summary of findings and quality of evidence

Supplementary table 5. Bolus fluids compared to maintenance fluids alone in children with severe febrile illness and any sign of impaired circulation (IC)

~~Table 4 and 5. Summary of findings and quality of evidence~~

For peer review only

For peer review only - http://bmjopen.bmj.com/site/about/guidelines.xhtml

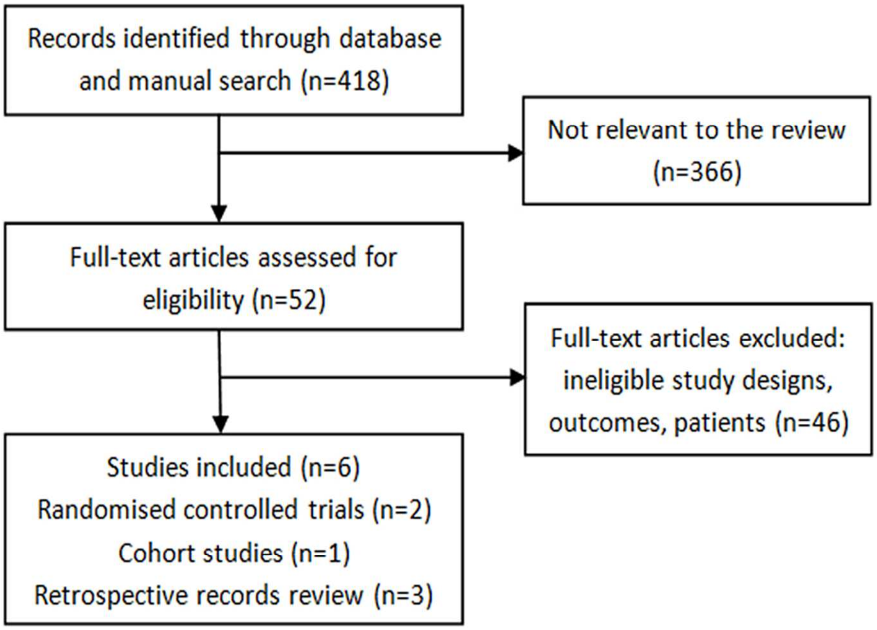

Figure 1. Results of literature search and studies selected
90x67mm (300 x 300 DPI)

For peer review only

Supplementary table 1. Characteristics of included studies

Study	Study design	Setting	Number of children	Intervention	Control	Co-interventions
Maitland 2011 [8]	RCT	Kenya Tanzania Uganda	3,141	20 to 40 mls/kg of 5% albumin or 0.9% saline over the first hour, followed by maintenance fluids	Intravenous maintenance fluids alone (2.5 to 4.0 mls/kg/hr)	Antibiotics, antimalarials, antipyretics, anticonvulant drugs, hypoglycemia treatment and blood transfusion
Santhanam 2008 [14]	RCT	India	147	20 to 40 mls/kg of Ringers lactate over 15 minutes followed by dopamine if therapeutic goal not achieved after 15 minutes	20 mls/kg over 20 minutes up to 60 mls/kg over 1 hour followed by dopamine if therapeutic goal not achieved at 1 hour	Antibiotics, anticonvulsant drugs, intubation, ventilation support, treatment of hypoglycemia, hypocalcemia and asthma
Carcillo 2009 [15]	Prospective cohort	USA	187 (a subgroup)	Received PALS/APLS - recommended resuscitation (20 to 40 mls/kg) or recovered quickly	Did not receive PALS/APLS - recommended resuscitation	Inotropic therapy
Han 2003 [17]	Retrospective records review	USA	91	Received PALS/APLS recommended resuscitation (>60 mls/kg normal saline) or recovered	Did not receive PALS/APLS recommended resuscitation	Antibiotics, inotropic/vasopressor support and hydrocortisone therapy
Carcillo 1991 [16]	Prospective cohort	USA	34	> 40 mls/kg total fluids (normal saline, Ringers lactate, 5% albumin or blood products)	<20 mls/kg total fluids or 20 to 40 mls/kg total fluids	Antibiotics, vasopressor and/or inotropic support and assisted ventilation
Oliveira 2008 [18]	Retrospective records review	Brazil	90	>40 mls/kg in the first hour (colloids, crystalloids, or packed red blood cells)	<20 mls/kg in the first hour	Antibiotics and inotropic support

For peer review only

Supplementary table 1. Characteristics of included studies (continued)

Study	Study design	Definition of severe febrile illness	Definition of circulatory impairment	Our classification	Overall mortality
Maitland 2011 [8]	Randomized controlled trial	Febrile illness with impaired consciousness and/or respiratory distress	One or more of: capillary refilling time ≥3 secs, severe tachycardia, temperature gradient or weak pulse	Some circulatory impairment (IC)<br><br>(Subgroup of severely impaired circulation (SIC))	9.5%<br><br>(42%)
Santhanam 2008 [14]	Randomized controlled trial	Children with septic shock	Tachycardia with signs of decreased perfusion including: decreased peripheral pulses compared with central pulses, altered alertness, flash capillary refill or capillary refill >2 secs, mottled or cool extremities, or decreased urine output	Some circulatory impairment (IC)	18%
Carcillo 2009 [15]	Prospective cohort	Infants and children with trauma and non-trauma diagnoses (including septic shock)	Prolonged capillary refill time (>3 seconds or mottled extremities) and/or hypotension (systolic blood pressure less than the fifth percentile for age according to PALS/APLS criteria)	Some circulatory impairment (IC)	12.5%
Han 2003 [17]	Retrospective records review	Infants and children with septic shock	Decreased perfusion, including decreased mental status, prolonged capillary refill time (>3 seconds), diminished peripheral pulses, or mottled extremities and hypotension (systolic blood pressure less than the fifth percentile for age)	Severe circulatory impairment (SIC)	29%
Carcillo 1991 [16]	Prospective cohort	Children with septic shock	Blood pressure <2 SDs below the mean for age, combined with three of the following four criteria for decreased perfusion: decreased peripheral pulses; mottled or cool extremities; tachycardia (heart rate >180 bpm for patients <5 years of age; and >160 bpm for patients ≥5 years of age); or urine output <1 mls/kg per hour (or <20 mls/hr in children weighing >20 kg)	Severe circulatory impairment (SIC)	51%
Oliveira 2008 [18]	Retrospective records review	Children with sepsis and septic shock	Decreased perfusion (decreased peripheral pulses, mottled or cool extremities, capillary refill time <1 second or >3 seconds), hypotension (systolic blood	Severe circulatory impairment (SIC)	47%

For peer review only

	pressure less than the fifth percentile for age, using the PALS formula), altered mental status, or oliguria (<1 mls/kg per hour)

bpm: Beats per minute; PALS: Pediatric Advanced Life Support; APLS: Advanced Pediatric Life Support; SD: Standard deviation;
IC: Severe febrile illness and any sign of impaired circulation;
SIC: Severe febrile illness and signs of severely impaired circulation;
IC but not SIC: Severe febrile illness and impaired circulation but not severely impaired circulation

For peer review only

Supplementary table 2. Summary of findings and quality of evidence

Bolus fluids compared to maintenance fluids alone in children with severe febrile illness and severely impaired circulation (SIC)

Patient or population: Children with severe febrile illness and severely impaired circulation
Settings: Hospitals (East Africa, USA, Brazil)
Intervention: Bolus fluid resuscitation (albumin or normal saline) followed by maintenance fluids
Comparison: Maintenance fluids alone[1]

Outcomes	Study design	Assumed risk	Corresponding risk (95% CI)	Relative effect (95% CI)	No of Participants (studies)	Quality of the evidence (GRADE)	Comments
		**Maintenance fluids alone**	**Bolus followed by maintenance fluids**				
**Mortality**	RCT	**20 per 100**	**48 per 100** (17 to 138)	**RR 2.40** (0.84 to 6.88)	65 (1 study)	⊕⊕⊖⊖ **Low**[2,3,4,5]	Maitland 2011 [8]
	Prospective cohort	**60 per 100**	**11 per 100** (2 to 73)	**RR 0.19** (0.03 to 1.21)	34 (1 study)	⊕⊖⊖⊖ **Very low**[6,7]	Carcillo 1991 [16]
	Retrospective cohort	**73 per 100**	**33 per 100** (NA)	**RR 0.45** (NA)	90 (1 study)	⊕⊖⊖⊖ **Very low**[8,9,10]	Oliveira 2008 [18]
	Retrospective cohort	**38 per 100**	**8 per 100** (2 to 30)	**RR 0.20** (0.05 to 0.78)	91 (1 study)	⊕⊖⊖⊖ **Very low**[11,12]	Han 2003 [17]

The **corresponding risk** (and its 95% confidence interval) is based on the assumed risk in the comparison group and the **relative effect** of the intervention (and its 95% CI).
CI: Confidence interval; **RR:** Risk ratio

GRADE Working Group grades of evidence
High quality: We are very confident that the true effect lies close to that of the estimate of the effect.
Moderate quality: We are moderately confident in the effect estimate: The true effect is likely to be close to the estimate of the effect, but there is a possibility that it is substantially different.
Low quality: Our confidence in the effect estimate is limited: The true effect may be substantially different from the estimate of the effect.
Very low quality: We have very little confidence in the effect estimate: The true effect is likely to be substantially different from the estimate of effect.

[1] All children received antibiotics, maintenance fluids and supportive care according to standard guidelines;
[2] This data represents a sub-group of patients from the large FEAST trial in East Africa [8]. These 65 patients had signs of a febrile illness and fulfilled the ETAT definition of shock;
[3] No serious risk of bias: Randomization and allocation concealment were adequate to reduce the risk of selection bias. Study staff were unblinded to the intervention;
[4] Downgraded by 1 for serious indirectness: Children with severe hypotension were excluded;
[5] Downgraded by 1 for serious imprecision: This subgroup of patients from the FEAST trial is severely underpowered to detect clinically important differences between the interventions;
[6] This study retrospectively examined health records of children with septic shock admitted to pediatric intensive care in Washington USA, and compares three groups receiving >40 mls/kg in the first hour, 20-40 mls/kg, and <20 mls/kg. Here we present > 40mls/ kg vs <40 mls/kg;
[7] Downgraded by 1 for serious imprecision: This trial is severely underpowered to confidently detect clinically important differences;
[8] This study retrospectively compared mortality in children with septic shock who received >40 mls/kg during the first hour of treatment with those receiving <20 mls/kg;
[9] Downgraded by 1 for serious indirectness: This study was conducted in a pediatric intensive care unit in Sao Paulo, Brazil, and over 80% had a severe pre-existing chronic disease such as malignancy;
[10] Downgraded by 1 for serious imprecision: The data were only presented as percentages and 95% CI could not be calculated. A P-value of <0.05 is stated by the authors;

For peer review only - http://bmjopen.bmj.com/site/about/guidelines.xhtml

[11] This study retrospectively examined the health records of children presenting with septic shock in the USA, requiring transport to a children hospital;
[12] Downgraded by 1 for risk of bias: This study compares 'appropriate fluid therapy' in line with ACCM/PALS guidelines with 'inadequate fluid therapy'. The 'appropriate therapy' group includes those where fluid was given in line with ACCM/PALS guidelines AND those who recovered quickly irrespective of how much fluid was given.

For peer review only

Supplementary table 3. Bolus fluids compared to maintenance fluids alone in children with severe febrile illness and severely impaired circulation (SIC)

Bolus fluids compared to maintenance fluids alone in children with severe febrile illness and any sign of impaired circulation (IC)

Quality assessment						Summary of findings				
						Number of patients		Effect size		
**Study design**	**Risk of bias**	**Inconsistency**	**Indirectness**	**Imprecision**	**Publication bias**	**No bolus**	**Bolus**	**Relative risk (95% CI)**	**Absolute effect**	**Quality**
**Outcome: Mortality**										
Randomised control trial‡ [8]	No serious risk of bias[1]	No serious inconsistency	Serious[2]	Serious[3]	Undetected	3/15 (20%)	24/50 (48%)	RR 2.40 (0.84 to 6.88)	28 more per 100 (from 3 fewer to 100 more)	⊕⊕⊖⊖ Low
Prospective cohort study† [16]	Serious[4]	No serious inconsistency	No serious indirectness	Serious[5]	Undetected	15/25 (60%)	1/9 (11%)	RR 0.19 (0.03 to 1.21)	49 fewer per 100 (from 58 fewer to 13 more)	⊕⊖⊖⊖ Very low
Retrospective cohort study¶ [18]	Serious[6]	No serious inconsistency	Serious[7]	Serious[8]	Undetected	73%††	33%††	RR 0.45 95% CI not estimatable	Not estimatable	⊕⊖⊖⊖ Very low
Retrospective cohort study‡‡ [17]	Serious[9]	No serious inconsistency	No serious indirectness	Serious[10]	Undetected	24/64 (37.5%)	2/27 (7.4%)	RR 0.20 (0.05 to 0.78)	30 fewer per 100 (from 8 fewer to 36 fewer)	⊕⊖⊖⊖ Very low

For peer re

CI: Confidence interval;

‡This data represents a sub-group of patients from the large FEAST trial in East Africa [8]. These 65 patients had signs of a febrile illness and fulfilled the ETAT definition of shock. The intervention comprised bolus fluid resuscitation (40 mls/kg albumin or normal saline) followed by maintenance fluids (2.5 to 4.0 mls/kg). The control (no bolus) group received maintenance fluids alone (2.5 to 4.0 mls/kg);

[1]No serious risk of bias: Randomization and allocation concealment were adequate to reduce the risk of selection bias. Study staff were unblinded to the intervention;

[2]Downgraded by 1 for serious indirectness: Children with severe hypotension were excluded;

[3]Downgraded by 1 for serious imprecision: This subgroup of patients from the FEAST trial is severely underpowered to detect clinically important differences between the interventions;

†This study retrospectively examined health records of children with septic shock admitted to pediatric intensive care in Washington USA, and compares three groups receiving >40 mls/kg in the first hour, 20-40 mls/kg, and <20 mls/kg. Here we present > 40mls/ kg vs <40 mls/kg;

[4]Adequate baseline characteristics were not presented;

[5]Downgraded by 1 for serious imprecision: This trial is severely underpowered to confidently detect clinically important differences;

¶This study retrospectively compared mortality in children with septic shock who received >40 mls/kg during the first hour of treatment with those receiving <20 mls/kg;

[6]Downgraded by 1 for risk of bias: The study compared survivors and non-survivors. The survivors were more likely to have had higher fluid volumes and were also significantly younger;

[7]Downgraded by 1 for serious indirectness: This study was conducted in a pediatric intensive care unit in Sao Paulo, Brazil, and over 80% had a severe pre-existing chronic disease such as malignancy;

[8]Downgraded by 1 for serious imprecision: The data were only presented as percentages and 95% CI could not be calculated. A P-value of <0.05 is stated by the authors;

†† A total of 45 children with septic shock died – however the number of deaths in the treatment groups was unclear (reported as 33%, >40 mls/kg and 73%, <20 mls/kg during the first hour of treatment);

‡‡ This study retrospectively examined the health records of children presenting with septic shock in the USA, requiring transport to a children hospital;

[9]Downgraded by 1 for risk of bias: This study compares 'appropriate fluid therapy' in line with ACCM/PALS guidelines with 'inadequate fluid therapy'. The 'appropriate therapy' group includes those where fluid was given in line with ACCM/PALS guidelines AND those who recovered quickly irrespective of how much fluid was given;

[10]Downgraded by 1 for serious imprecision: Small sample size (N=91 patients). Small number of events (N=26 deaths).

Patient or population: Children with severe febrile illness and some circulatory impairment
Settings: Hospitals (East Africa, India, USA)
Intervention: Bolus fluid resuscitation (albumin or normal saline) followed by maintenance fluids
Comparison: Maintenance fluids alone[1]

Outcomes	Study design	Assumed risk	Corresponding risk (95% CI)	Relative effect (95% CI)	No of Participants (studies)	Quality of the evidence (GRADE)	Comments
		Maintenance fluids alone	Bolus followed by maintenance fluids				
**Mortality** at 48 hours	RCT	**73 per 1000**	**106 per 1000** (82 to 135)	**RR 1.45** (1.13 to 1.86)	3,141 (1 study)	⊕⊕⊕⊕ **High**[2,3,4,5,6]	Maitland 2011 [8]
**Mortality** at 72 hours	RCT	**18 per 100**	**18 per 100** (9 to 35)	**RR 0.99** (0.49 to 1.98)	147 (1 study)	⊕⊕⊖⊖ **Low**[7,8,9]	Santhanam 2008 [14]
**Mortality**	Prospective cohort	**14 per 100**	**12 per 100** (5 to 27)	**RR 0.84** (0.37 to 1.91)	187 (1 study)	⊕⊖⊖⊖ **Very low**[10,11]	Carcillo 2009 [15]
**Mortality** at 4 weeks	RCT	**87 per 1000**	**121 per 1000** (97 to 152)	**RR 1.39** (1.11 to 1.74)	3,141 (1 study)	⊕⊕⊕⊕ **High**[4,5,6]	Maitland 2011 [8]
**Neurologic sequelae** at 4 weeks	RCT	**20 per 1000**	**21 per 1000** (12 to 35)	**RR 1.03** (0.61 to 1.75)	2,983 (1 study)	⊕⊕⊕⊖ **Moderate**[4,5,12]	Maitland 2011 [8]
**Pulmonary edema, increased intracranial pressure**	RCT	**16 per 1000**	**24 per 1000** (14 to 41)	**RR 1.46** (0.85 to 2.53)	3,141 (1 study)	⊕⊕⊕⊖ **Moderate**[4,5,12]	Maitland 2011 [8]
**Severe anaemia:** mortality at 48 hours	RCT	**90 per 1000**	**155 per 1000** (105 to 227)	**RR 1.71** (1.16 to 2.51)	987 (1 study)	⊕⊕⊕⊖ **Moderate**[13]	Maitland 2011 [8]
**Non-severe anaemia:** mortality at 48 hours	RCT	**6 per 100**	**8 per 100** (6 to 12)	**RR 1.31** (0.93 to 1.84)	2,067 (1 study)	⊕⊕⊕⊖ **Moderate**[14]	Maitland 2011 [8]

The **corresponding risk** (and its 95% confidence interval) is based on the assumed risk in the comparison group and the **relative effect** of the intervention (and its 95% CI).
CI: Confidence interval; **RR:** Risk ratio

GRADE Working Group grades of evidence
High quality: We are very confident that the true effect lies close to that of the estimate of the effect.
Moderate quality: We are moderately confident in the effect estimate: The true effect is likely to be close to the estimate of the effect, but there is a possibility that it is substantially different.
Low quality: Our confidence in the effect estimate is limited: The true effect may be substantially different from the estimate of the effect.
Very low quality: We have very little confidence in the effect estimate: The true effect is likely to be substantially different from the estimate of effect.

[1] All children received antibiotics, maintenance fluids and supportive care according to standard guidelines;
[2] The FEAST inclusion criteria were: One or more of: capillary refill >2 secs, lower limb temp gradient, weak pulse, tachycardia;
[3] The bolus was initially 20 mls/kg over 1 hour but this was increased to 40 mls/kg part-way through the trial following a preliminary analysis which showed increased mortality with the bolus;
[4] No serious risk of bias: Randomization and allocation concealment were adequate to reduce the risk of selection bias. Study staff were unblinded to the intervention;

only

[5] No serious indirectness: Children with severe hypotension, severe malnutrition, gastroenteritis, or shock due to trauma surgery or burns were excluded;
[6] No serious imprecision: The result is clinically important and statistically significant;
[7] Children with septic shock in hospital in India were randomized to 20 to 40 mls/kg over 15 mins or 20 mls/kg over 20 mins up to a maximum of 60 mls/kg over an hour;
[8] Downgraded by 1 for serious indirectness: The comparison in this trial is not easily related to the comparison in FEAST;
[9] Downgraded by 1 for serious imprecision: The trial is underpowered to confidently rule out differences;
[10] This non-randomized study compares children with shock who received resuscitation in line with PALS guidelines early, and those that didn't. This subgroup excludes trauma cases, but includes cardiac, neurological, respiratory, sepsis and gastroenteritis as causes of shock;
[11] Downgraded by 1 for serious indirectness as the fluid volumes administered are not presented;
[12] Downgraded by 1 for serious imprecision: The 95% CI includes a relative risk of 1 (no effect) and appreciable benefit and harm;
[13] Severe anaemia, hemoglobin <5 g/dl: Downgraded by 1 for serious imprecision (95% CI includes appreciable harm) and few events (n=131 deaths);
[14] Non-severe anaemia, hemoglobin ≥5 g/dl: Downgraded by 1 for serious imprecision (95% CI includes 1 (no effect)) and few events (n=157 deaths).

For peer review only

Supplementary table 5. Bolus fluids compared to maintenance fluids alone in children with severe febrile illness and any sign of impaired circulation (IC)

Quality assessment						Summary of findings				
						Number of patients		Effect size		
**Outcomes Study design**	**Risk of bias**	**Inconsistency**	**Indirectness**	**Imprecision**	**Publication bias**	**No bolus**	**Bolus**	**Relative risk (95% CI)**	**Absolute effect**	**Quality**
Mortality at 48 hours, RCT‡ [8]	No serious risk of bias[1]	No serious inconsistency	No serious indirectness[2]	No serious imprecision[3]	Undetected	76/1044 (7.3%)	221/2097 (10.5%)	RR 1.45 (1.13 to 1.86)	33 more per 1000 (from 9 more to 63 more)	⊕⊕⊕⊕ High
Mortality at 72 hours, RCT† [14]	No serious risk of bias[4]	No serious inconsistency	Serious[5]	Serious[6]	Undetected	13/73 (17.8%)	13/74 (17.6%)	RR 0.99 (0.49 to 1.98)	0 fewer per 100 (from 9 fewer to 17 more)	⊕⊕⊖⊖ Low
Mortality Prospective cohort ¶ [15]	Serious[7]	No serious inconsistency	Serious[8]	Serious[9]	Undetected	18/128 (14.1%)	7/59 (11.9%)	RR 0.84 (0.37 to 1.91)	2 fewer per 100 (from 9 fewer to 13 more)	⊕⊖⊖⊖ Very low
Mortality at 4 weeks, RCT [8]	No serious risk of bias[1]	No serious inconsistency	No serious indirectness[2]	No serious imprecision[3]	Undetected	91/1044 (8.7%)	254/2097 (12.1%)	RR 1.39 (1.11 to 1.74)	34 more per 1000 (from 10 more to 65 more)	⊕⊕⊕⊕ High
Neurologic sequelae at 4 weeks, RCT [8]	No serious risk of bias[1]	No serious inconsistency	No serious indirectness[2]	Serious[10]	Undetected	20/997 (2.0%)	41/1986 (2.1%)	RR 1.03 (0.61 to 1.75)	1 more per 1000 (from 8 fewer to 15 more)	⊕⊕⊖⊖ Moderate
Pulmonary edema, increased intracranial pressure, RCT [8]	No serious risk of bias[1]	No serious inconsistency	No serious indirectness[2]	Serious[10]	Undetected	17/1044 (1.6%)	50/2097 (2.4%)	RR 1.46 (0.85 to 2.53)	7 more per 1000 (from 2 fewer to 25 more)	⊕⊕⊖⊖ Moderate
Severe anaemia: mortality at 48 hours, RCT [8]	No serious risk of bias[1]	No serious inconsistency	No serious indirectness[2]	Serious[11]	Undetected	30/332 (9%)	101/655 (15.4%)	RR 1.71 (1.16 to 2.51)	64 more per 1000 (from 14 more to 136 more)	⊕⊕⊖⊖ Moderate
Non-severe anaemia: mortality at 48 hours, RCT [8]	No serious risk of bias[1]	No serious inconsistency	No serious indirectness[2]	Serious[12]	Undetected	43/683 (6.3%)	114/1384 (8.2%)	RR 1.31 (0.93 to 1.84)	20 more per 1000 (from 4 fewer to 53 more)	⊕⊕⊖⊖ Moderate

For peer review only

RCT: Randomised controlled trial;
‡Children were randomized to a fluid bolus (20 to 40 mls/kg albumin or normal saline over 1 hour) or maintenance fluids (2.5 to 4.0 mls/kg/hour);
[1]No serious risk of bias: Randomization and allocation concealment were adequate to reduce the risk of selection bias. Study staff were unblinded to the intervention;
[2]No serious indirectness: Children with severe hypotension, severe malnutrition, gastroenteritis, or shock due to trauma surgery or burns were excluded;
[3]No serious imprecision: The result is clinically important and statistically significant;
†Children with septic shock in hospital in India were randomized to 20 to 40 mls/kg over 15 mins or 20 mls/kg over 20 mins up to a maximum of 60 mls/kg over an hour;
[4]Low risk of selection bias (random sequence generation and allocation concealment adequate); low risk of reporting bias;
[5]Downgraded by 1 for serious indirectness: The comparison in this trial is not easily related to the comparison in FEAST trial [8];
[6]Downgraded by 1 for serious imprecision: The trial is underpowered to confidently rule out differences;
¶ This non-randomized study compares children with shock who received resuscitation in line with PALS guidelines early, and those that didn't. This subgroup excludes trauma cases, but includes cardiac, neurological, respiratory, sepsis and gastroenteritis as causes of shock;
[7]High risk for selection bias and confounding: 'Received recommended APLS/PALS fluid therapy' was defined as those who recovered regardless of fluid therapy plus those who didn't recover but received >20 mg/kg of fluids. Children who didn't receive recommended APLS/PALS treatment were significantly younger and had significantly longer capillary refill times, lower blood pressure, and higher oxygen requirements;
[8]Downgraded by 1 for serious indirectness as the fluid volumes administered are not presented;
[9]Downgraded by 1 for serious imprecision: Small sample size (N=187 patients) and small number of events (N=25 deaths due to sepsis);
[10]Downgraded by 1 for serious imprecision: The 95% CI includes a relative risk of 1 (no effect) and appreciable benefit and harm;
[11]Severe anaemia, hemoglobin <5 g/dl: Downgraded by 1 for serious imprecision (95% CI includes appreciable harm) and few events (n=131 deaths);
[12]Non-severe anaemia, hemoglobin ≥5 g/dl: Downgraded by 1 for serious imprecision (95% CI includes 1 (no effect)) and few events (n=157 deaths).

review only

PRISMA 2009 Checklist

Section/topic	#	Checklist item	Reported on page #
**TITLE**			
Title	1	Identify the report as a systematic review, meta-analysis, or both.	1
**ABSTRACT**			
Structured summary	2	Provide a structured summary including, as applicable: background; objectives; data sources; study eligibility criteria, participants, and interventions; study appraisal and synthesis methods; results; limitations; conclusions and implications of key findings; systematic review registration number.	2-3
**INTRODUCTION**			
Rationale	3	Describe the rationale for the review in the context of what is already known.	5
Objectives	4	Provide an explicit statement of questions being addressed with reference to participants, interventions, comparisons, outcomes, and study design (PICOS).	6
**METHODS**			
Protocol and registration	5	Indicate if a review protocol exists, if and where it can be accessed (e.g., Web address), and, if available, provide registration information including registration number.	No protocol available
Eligibility criteria	6	Specify study characteristics (e.g., PICOS, length of follow-up) and report characteristics (e.g., years considered, language, publication status) used as criteria for eligibility, giving rationale.	6
Information sources	7	Describe all information sources (e.g., databases with dates of coverage, contact with study authors to identify additional studies) in the search and date last searched.	6
Search	8	Present full electronic search strategy for at least one database, including any limits used, such that it could be repeated.	6
Study selection	9	State the process for selecting studies (i.e., screening, eligibility, included in systematic review, and, if applicable, included in the meta-analysis).	6
Data collection process	10	Describe method of data extraction from reports (e.g., piloted forms, independently, in duplicate) and any processes for obtaining and confirming data from investigators.	6-7
Data items	11	List and define all variables for which data were sought (e.g., PICOS, funding sources) and any assumptions and simplifications made.	7
Risk of bias in individual studies	12	Describe methods used for assessing risk of bias of individual studies (including specification of whether this was done at the study or outcome level), and how this information is to be used in any data synthesis.	7
Summary measures	13	State the principal summary measures (e.g., risk ratio, difference in means).	7

For peer review only

PRISMA 2009 Checklist

Synthesis of results	14	Describe the methods of handling data and combining results of studies, if done, including measures of consistency (e.g., I^2) for each meta-analysis.	7

Section/topic	#	Checklist item	Reported on page #
Risk of bias across studies	15	Specify any assessment of risk of bias that may affect the cumulative evidence (e.g., publication bias, selective reporting within studies).	7
Additional analyses	16	Describe methods of additional analyses (e.g., sensitivity or subgroup analyses, meta-regression), if done, indicating which were pre-specified.	7
**RESULTS**			
Study selection	17	Give numbers of studies screened, assessed for eligibility, and included in the review, with reasons for exclusions at each stage, ideally with a flow diagram.	7-8
Study characteristics	18	For each study, present characteristics for which data were extracted (e.g., study size, PICOS, follow-up period) and provide the citations.	8, Supplementary table 1
Risk of bias within studies	19	Present data on risk of bias of each study and, if available, any outcome level assessment (see item 12).	8, tables 3, Supplementary tables 2-5
Results of individual studies	20	For all outcomes considered (benefits or harms), present, for each study: (a) simple summary data for each intervention group (b) effect estimates and confidence intervals, ideally with a forest plot.	8-10; Supplementary tables 2-5
Synthesis of results	21	Present results of each meta-analysis done, including confidence intervals and measures of consistency.	8-10;
Risk of bias across studies	22	Present results of any assessment of risk of bias across studies (see Item 15).	8-10; Supplementary tables 2-5
Additional analysis	23	Give results of additional analyses, if done (e.g., sensitivity or subgroup analyses, meta-regression [see Item 16]).	None.
**DISCUSSION**			
Summary of evidence	24	Summarize the main findings including the strength of evidence for each main outcome; consider their relevance to key groups (e.g., healthcare providers, users, and policy makers).	10-11
Limitations	25	Discuss limitations at study and outcome level (e.g., risk of bias), and at review-level (e.g., incomplete retrieval of identified research, reporting bias).	10-11

For peer review only

PRISMA 2009 Checklist

Conclusions	26	Provide a general interpretation of the results in the context of other evidence, and implications for future research.	10-11
**FUNDING**			
Funding	27	Describe sources of funding for the systematic review and other support (e.g., supply of data); role of funders for the systematic review.	12

From: Moher D, Liberati A, Tetzlaff J, Altman DG, The PRISMA Group (2009). Preferred Reporting Items for Systematic Reviews and Meta-Analyses: The PRISMA Statement. PLoS Med 6(6): e1000097. doi:10.1371/journal.pmed1000097

For more information, visit: **www.prisma-statement.org**.

For peer review only

For peer review only - http://bmjopen.bmj.com/site/about/guidelines.xhtml
